# Recent advanced applications of metasurfaces in multi-dimensions

**DOI:** 10.1515/nanoph-2022-0803

**Published:** 2023-02-24

**Authors:** Quan Yuan, Qin Ge, Linsen Chen, Yi Zhang, Yuhang Yang, Xun Cao, Shuming Wang, Shining Zhu, Zhenlin Wang

**Affiliations:** National Laboratory of Solid State Microstructures, School of Physics, School of Electronic Science and Engineering, Nanjing University, Nanjing 210093, China; National Laboratory of Solid State Microstructures, School of Physics, Nanjing University, Nanjing 210093, China; Collaborative Innovation Center of Advanced Microstructures, Nanjing 210093, China

**Keywords:** application, dimension, metasurface, wavelength scale

## Abstract

Unlike traditional optical components, which rely on the gradual accumulation of light along the optical path over a distance much larger than the wavelength to form a wavefront, metasurfaces manipulate light field properties on the wavelength thickness by specially arranging various meta-atoms. Due to the ease of integration and compact planar structure, metasurfaces play a key role in the light field manipulations. Here, we review the recent advances of metasurfaces in multi-dimensions, including light wavelength, polarization, orbital angular momentum(OAM), and angular response. Progress in these fields has brought new applications in areas such as imaging, display, communication, and information encryption, etc. Finally, we also discuss the challenges and prospects of metasurfaces applications.

## 1 Introduction

Light is the main medium for humans to observe the world, and a large amount of information comes from visuals through human eyes. The development of optical engineering has enabled human beings to break through the limitations of their own senses. No longer limited to what is visible to the naked eye, humans can observe information by making optical devices from small cells to the large universe. Traditional optical devices rely on light traveling over distances much larger than the wavelength to form a wavefront. In this way, substantial changes in the amplitude, phase, or polarization of the light waves are gradually accumulated along the optical path. However, the accumulation of slow changes in optical parameters leads to the realization of various optical functions that often require bulky and complex optical devices, which poses challenges for processability, portability, and cost. Metasurfaces are two-dimensional artificial electromagnetic materials composed of a specific arrangement of subwavelength optical elements called meta-atoms that allow complete control of the electromagnetic wavefront [1–19]. Due to its compact planar structure and customized design capabilities, metasurface optical devices can meet the development requirements for higher portability and integration. At the same time, the compatibility with complementary metal-oxide-semiconductor (CMOS) in manufacturing makes metasurfaces get the potential for practical applications.

There are many kinds of metasurface phase manipulation including the resonance phase, transmission phase, and geometric phase. The resonant phase is a localized surface plasmon resonance generated by free electron oscillations in the subwavelength metallic unit. By designing metal metasurfaces of different shapes [20–23], it is possible to achieve subwavelength-scale light manipulation. The propagating phase has been studied by exploiting the effective refractive index to control the phase delay. The phase of light propagation can be tuned by changing the volume ratio, aspect ratio, and height of meta-atoms [24]. The geometric phase, also known as the Pancharatnam–Berry (PB) phase, is to endow the anisotropic metasurface atoms with phase retardation proportional to their rotation angle [25, 26]. With these manipulation methods, metasurfaces can achieve special control of the degrees of freedom of light such as amplitude [27–29], phase [30–33], frequncy [34], and polarization [35–37] on the subwavelength scale.

Through arbitrary light tailoring, metasurfaces can be used as key optical devices in different application scenarios. In this review, we summarize the applications realized by metasurfaces in recent years based on the control of different degrees of freedom of light, and looks forward to more application space for metasurfaces in the future. There have been reviews discussing areas like dispersion engineering [38], meta-waveguide [39], nano-optical sensors [40], nanoprinting [41], and tunable metasurface [42]. There are also reviews that classificate from materials and manufacturing processes [43]. However, different from these, we discuss recent metasurface applications through four dimensions of manipulation. In the second section, we summarize the research on wavelength tuning by metasurfaces, including recent work in the fields of metalens, structural color, spectral imaging, and color routers. In Section 3, we mainly conclude the metasurfaces for polarization applications, including holographic multiplexing, encryption, vector beams, and polarization imaging. In the fourth section, we summarize the research on the angular momentum of metasurfaces, including optical communication, optical force, etc. The research on the angular momentum of light is also a research hotspot in recent years. In the fifth section, we introduce the application scenarios brought about by the multiplexing of the direction and angle of the incident light. In the last section, we give a summary and an outlook on future applications of metasurfaces.

## 2 Wavelength-dependent metasurface

Dispersion is an important property of optical materials, which represents the response function of the different wavelengths of light interacting with matter. In imaging systems, dispersion effects usually cause chromatic aberration, which seriously affects image quality. Traditional optics integrates several materials with complementary dispersion into a single component to obtain the same focal length at multiple wavelengths, achieving the elimination of chromatic aberration. However, this approach adds weight, complexity, and cost to the optical imaging system. In contrast, wavefront control via metasurfaces could provide a thin, light, and flexible approach to dispersion engineering.

In previous work, achromatism at three discrete wavelengths in the mid-infrared [44] and at communication wavelengths [45] has been achieved through dielectric nanopillars with specific rotation angles. Achromatic metalens with a bandwidth of 60 nm has been successfully demonstrated [46]. However, 60 nm bandwidth achromatism is still too narrow for practical applications. Wang et al. [47] divided the desired phase into wavelength-independent and wavelength-dependent terms. Through the geometric phase, a dispersion-independent phase is obtained, and the dispersion of different incident wavelengths is manipulated through the resonant phase of the metal nanostructure. Finally, continuous achromatic focusing over a broadband range of 1200–1680 nm was demonstrated. Furthermore, Wang et al. [48] achieved a transmission broadband achromatic imaging in the visible light range of 400–660 nm through GaN nanostructures, with an average efficiency of 40% and good image quality. Chen et al. demonstrated a broadband focusing lens that performs achromatic focusing in the wavelength range of 470–670 nm and achieves high-quality white light imaging [49].

**Figure 1:**
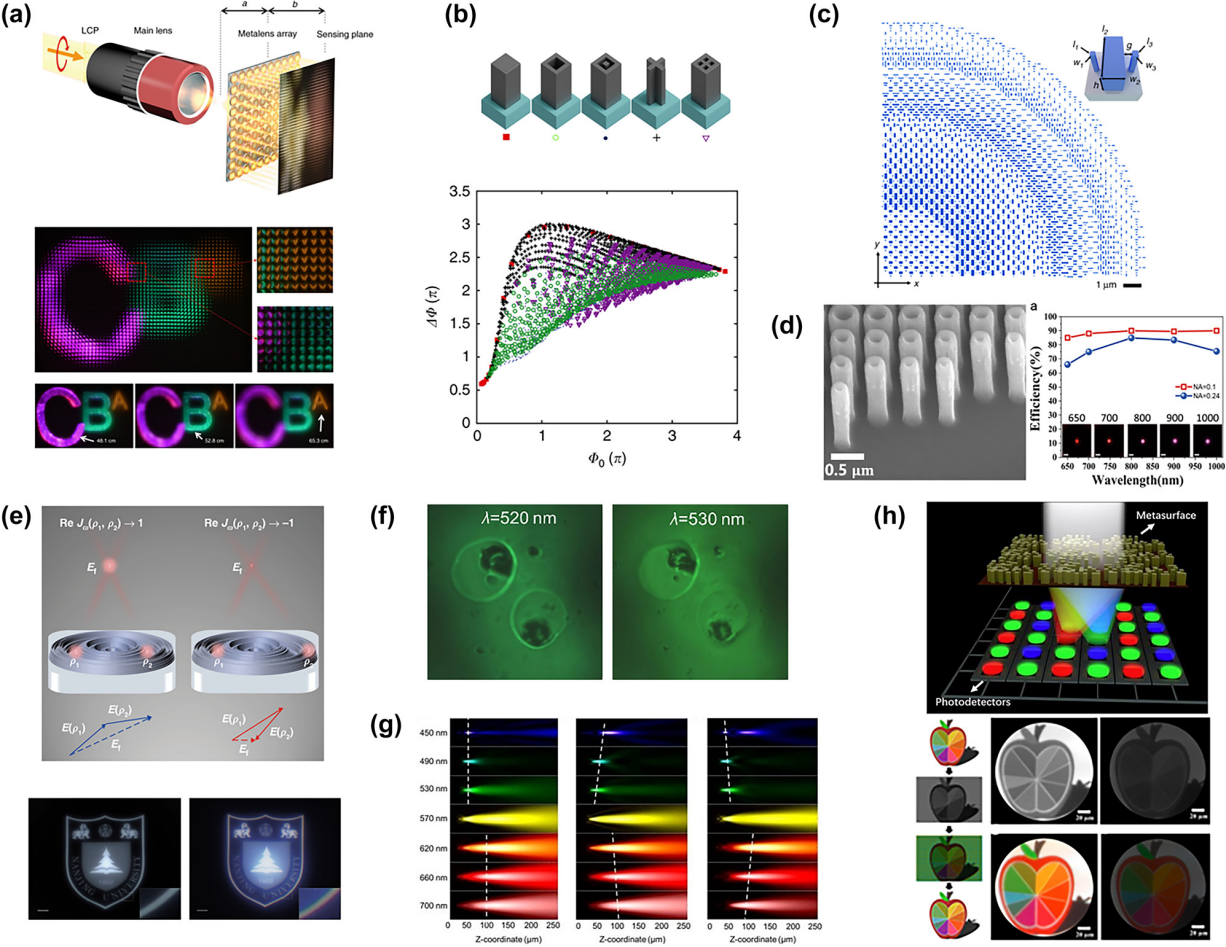
Metasurface optics with dispersion control. (a) Schematic diagram of light-field imaging with metalens array and rendered images. And the characteristics of the radiance captured by the focused metalens array light-field-imaging system, below show rendered images with different focusing depths [50]. (b) Meta-unit archetypes and calculated phase and dispersion for four-fold symmetry [52]. (c) The layout of a quadrant of the metalens, the inset shows a schematic diagram of its constituent elements [53]. (d) The corresponding tilt-view SEM image of the metalens. And the numerically calculated efficiencies of TiO_2_ metalenses at different wavelengths [54]. (e) Schematics of the coherence of wave distortion in MDL [55]. An image of the Nanjing University logo taken from a sample and a refractive lens, scale bars, 2 mm. (f) Microscopic spectral tomography of frog egg cells. At *λ* = 520 nm and 530 nm show the clearest image of the cell membrane and nucleus, respectively [56]. (g) Simulation results. Discrete bandwidth regions (450–530 nm and 620–700 nm) in the visible spectrum appear different dispersive properties [57]. (h) Schematic of the metasurface-based Bayer-type color router (MBCR). And below shows the comparison of imaging response between MBCR and Bayler color filters (BCFs) [58].

Since the compensation phase required for achromatic is a function of the lens diameter, the maximum size of the lens is limited by the maximum compensable phase, which can be described by the formula *R*_max_NAΔ*w* ≤ 2*c*Δ*ϕ*. Wang et al. designed a light-field camera with an achromatic metalens array (Figure 1a). Using large-area achromatic light field imaging to solve the problem that a single achromatic metalens cannot enlarge the diameter. And for the first time, it breaks through the problem that broadband achromatism cannot be realized in traditional light field imaging. Realize high efficiency, high numerical aperture (NA), and high-resolution 3D light field imaging [50]. Fan et al. also designed an achromatic metalens array. And achieved 430–780 nm achromatic light field imaging [51].

The use of geometric phase causes the achromatic imaging metalens to work only under circularly polarized illumination. How to realize the polarization-insensitive achromatic lens has attracted people’s attention. Shrestha et al. [52] designed metasurfaces using nanopillars with 4-fold rotational symmetry to achieve broadband achromatic focusing in arbitrary polarization states (Figure 1b). By using solid, hollow, concentric, X-shaped, and square pillars as meta-atoms and creating a cell phase response database that traverses all tunable shape parameters. They designed a NIR broadband achromatic un-polarized metalens. Chen et al. subsequently proposed the use of anisotropically shaped finned nanorods to achieve polarization-insensitive achromatic focusing for more accurate tuning of relative phase, group delay, and group delay dispersion. By constructing the fin dispersion space with a limited rotation angle, the achromatic focusing with the focusing efficiency of incident light with a full polarization state at 460–700 nm is realized (Figure 1c) [53]. Wang et al. achieved high-quality polarization-insensitive achromatic metasurface. Using a top–down fabrication technique, they fabricated symmetric TiO_2_ nanopillars with record aspect ratios. Their work achieved an achromatic focusing lens with an average biological window efficiency of 77.1%–88.5% and a numerical aperture of 0.24–0.1, comparable to the image quality and resolution recorded by commercial objectives in biological imaging (Figure 1d) [54]. Xiao et al. designed an achromatic multilevel diffractive lens (AMDL) to break through the limiting relationship between the size and NA of the metalens. They show that the performance limitation of non-ideal lenses is due to a reduction in coherence, and develop a frequency-domain coherence function to characterize it. Based on this principle, they optimized the manufacture of AMDLs with a diameter of 1 cm and a thickness of 15 μm in an ultra-wide wavelength range (400–1100 nm), which has extremely high comprehensive performance and broad application prospects in conventional imaging systems (Figure 1e) [55].

Metasurfaces can also utilize natural dispersion or manipulate dispersion to obtain novel chromatic optical distributions. Chen et al. designed aplanatic metalens to eliminate the spherical aberration under large NA imaging for the incidence of spherical waves in microscopic imaging. And provided high longitudinal resolution by using the large diffractive dispersion of the metalens. Finally, a tomographic imaging system with spectral focus tuning and optical zooming is fabricated. The system is capable of achieving a 42% focal length change in the 450–660 nm wavelength range, allowing individual imaging at different rotation angles on slides at different depths, and clearly imaging the cell membrane and nucleus of biological cells in experiments (Figure 1f) [56]. Sisler et al. used paired TiO_2_ fin nanopillars and designed multifunctional dispersion control by PB phase. In order to design different dispersion properties at different bands, the group delay and group delay dispersion of each fin cell need to be tuned to corresponding values at the upper and lower boundaries of the band. Multifunctional dispersion has been realized through different focal lengths in the range of 450–530 nm and 620–700 nm (Figure 1g) [57].

Traditionally, color filters are polymer filters on each pixel which is called the Bayer filter. These filters allow only one color of light to pass through each pixel location, while other colors are absorbed or reflected. As a result, at least 2/3 of the light energy is lost at each pixel position, and thus the imaging quality has always been affected by the loss of transmission. When the pixel size becomes small, the optical crosstalk between pixels becomes significant due to the presence of small absorption coefficients of organic dyes. By employing an inverse-design method, Zou et al. [58] demonstrate a pixel-level metasurface-based Bayer-type color router, which presents peak color collection efficiencies far higher than the ideal efficiency of traditional filter film for red, green, and blue light. The metasurface divides 4 beams of light into a cycle and focuses on 2 × 2 pixels of red, green, green, and blue. Such a design can be directly plugged into existing sensors without the need for subsequent color conversion algorithms to convert. They used the color router for imaging and compared its quality with traditional color filters. The imaging brightness was greatly improved with very high fidelity (Figure 1h). Miyata et al. also used polarization-insensitive metalens instead of a color filter to classify the primary colors of high-density pixels to achieve a high-sensitivity color image sensor without sacrificing image quality, incidence angle tolerance, or spatial resolution. Combining the metalens and filter architectures used in the study further improves light detection and color purity [59].

It is different from achromatic white light imaging which only pursues spatial intensity information. The hyperspectral image contains both the spatial and spectral information of the shooting scene, and the information capture of the spectral dimension can more accurately detect the physical characteristics of the target. Therefore, hyperspectral is widely used in food safety, medical detection, remote sensing imaging, geological exploration, and other fields. However, traditional spectrometers are bulky and need to sacrifice temporal and spatial resolution for higher spectral resolution, which is not conducive to integration and miniaturization. The planarization and high compactness of metasurfaces have important advantages in the field of spectral imaging, which can achieve higher spatial and spectral resolution while pursuing smaller devices.

**Figure 2:**
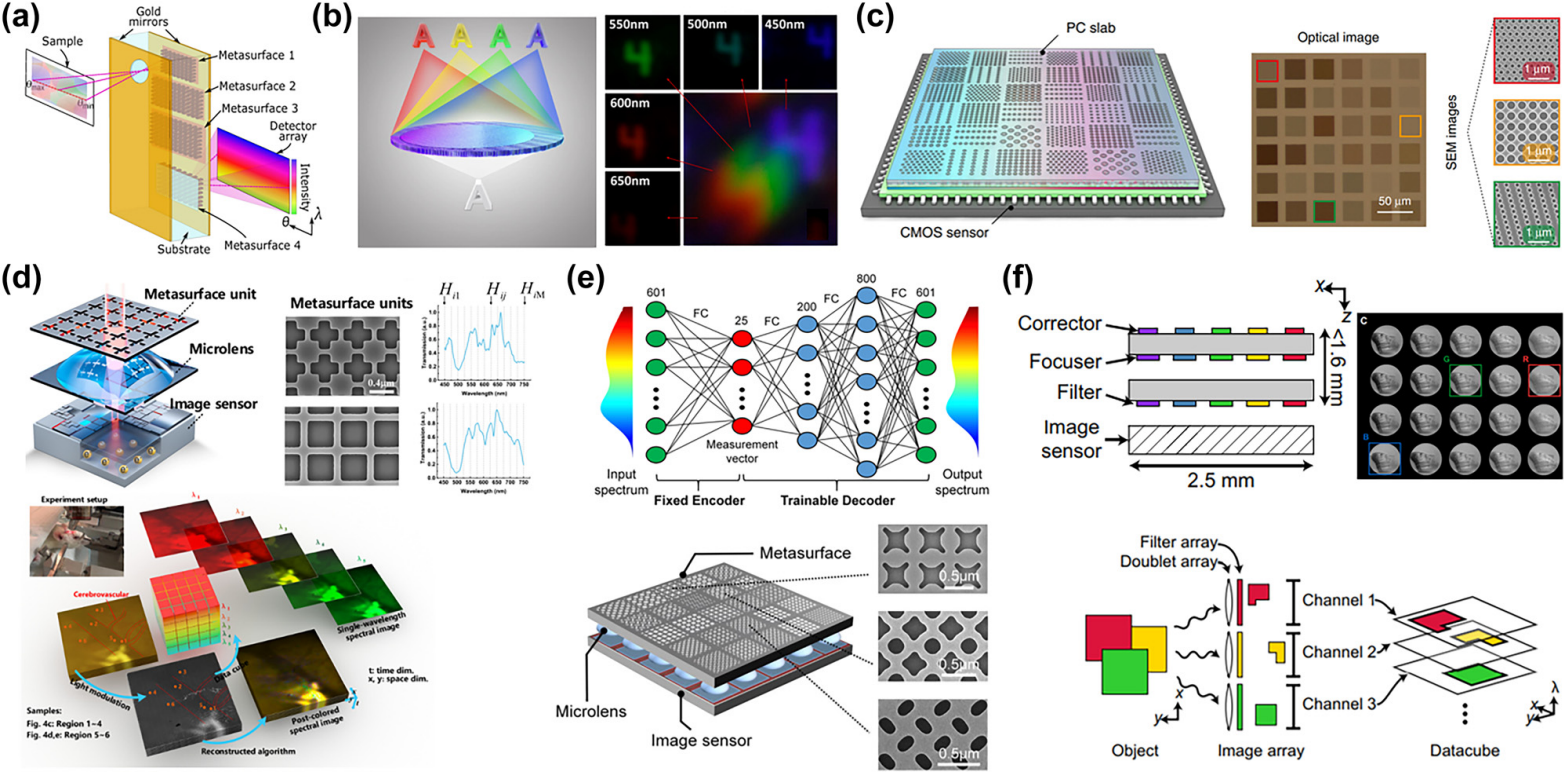
Application of metasurface in spectrometer direction. (a) Schematic diagram of push broom foldable hypersurface spectrometer [60]. (b) Sketch map of transversely dispersive metalens. Using white light illumination with a transmission window from 450 to 650 nm, the metalens’ transverse dispersion made the letter “4” imaged at different positions at different wavelengths [62]. (c) PC slabs with different parameters on top of a CMOS sensor array consist the spectrometer, an optical image of the fabricated 6 × 6 PC structures, and three selected scanning electron microscopy (SEM) images [63]. (d) Schematic diagram of basic modulation unit super surface unit; SEM image and spectral modulation action curve; computed reconstruction of 5 single wave adult rat cerebrovascular images in 601 bands [64]. (e) The spectrometer consists of a super surface layer, a microlens layer, and an image sensor layer with 400 unique free shape super atoms; schematic diagram of spectrum encoder-decoder network: the connection weight of the encoder corresponds to the transmission spectrum of the super surface unit; decoder can be trained for spectrum reconstruction [65]. (f) MSSI contains an aligned array of metasurface bandpass filters and doublet lenses comprising two metasurfaces, filters discriminate light traversing the doublet lenses into spectral channels; every channel is registered to form a 3D spectral datacube; normalized images of 20 butterfly wings [66].

Based on the principle of spectroscopic, Dana et al. [60] designed a linear scanning foldable spectrometer based on the metasurface, with a spectral resolution of 750 nm–850 nm, a spectral resolution of 1.5 nm, and an angular resolution of 0.075° (Figure 2a). There are four metasurfaces on the same side of the 1 mm thick fused silicon substrate to disperse and focus light of different wavelengths. The gold mirrors on both sides of the designed structure reflected the light many times, and the optical path from the dispersive metasurface to the CCD was increased with a more compact structure. According to the principle of a push-broom hyperspectral imager, Billuart et al. [61] conducted numerical simulation on a single metasurface, and achieved spectral dispersion and focusing through dispersion engineering of a two-stage nanopillars library. The spectral resolution is 8.5 nm, and the field of view around the vertical incidence angle is 8° (angular resolution is 0.2°). Hua et al. [62] realized the ultra-compact spectral light-field imaging (SLIM) by combining a transversely dispersive metalens and a monochromatic imaging sensor. Code sub-apertures separate images and serve as prior knowledge for reconstruction. Combined with the dispersion reconstruction algorithm, an imaging effect with a spectral resolution of 4 nm and a spatial resolution close to the diffraction limit can be obtained in one snapshot (Figure 2b). For the first time, 3D spatial information and additional spectral information can be simultaneously captured in the same device.

Dispersive spectral cameras need sufficient spatial distribution to accommodate the broadening of spectral information. With the development of computational optics, it is possible to reconstruct the desired spectral image with less effective information through calculation. Because it is unnecessary to obtain all the information of the spectral 3D data cube, it has fast speed and less redundant information. Wang et al. [63] adjusted the transmission spectra of different regions by changing the period, lattice constant and hole size of photonic crystals (PCs), and realized a random filtering spectrometer with a resolution of 1 nm in the 550–750 nm optical band (Figure 2c). Periodic nanoelement atoms coupled the incident light from free space to the transverse propagation mode and reflected it back and forth, finally focusing on the imaging plane 1 mm away from the metasurface array. Lee et al. [67] integrated the dielectric multilayer filter into the CMOS image sensor and adjusted the transmission wavelength of the corresponding spectral channel by changing the size of the array of Si atoms embedded in the corresponding pixel multilayer. The near-infrared spectral imager with a spectral resolution of 2.0 nm was successfully demonstrated. Hyperspectral images are obtained by overlaying images of a different wavelength.

Wu et al. [68] designed a random all-dielectric metasurface filter, combined with the compression sensing algorithm, to reconstruct the incident spectrum in the visible light range with the minimum full width at half maximum (FWHM) of 4.8 nm. Xiong et al. [64] demonstrated single-shot hyperspectral imaging (Figure 2d). They designed a reconfigurable metasurface based on image adaptation, through the spatial multiplexing of the unit structure, combined with the compressive sensing algorithm, a spatial resolution of more than 150,000 pixels and a spectral resolution of 0.8 nm are simultaneously achieved. For the first time, a real-time hyperspectral imaging chip was successfully fabricated. In addition to spectral image reconstruction, the algorithm can also be used for metasurface design. Yang et al. [65] believed that regular shape meta-atoms limited the further improvement of spectral imaging performance (Figure 2e). Therefore, the algorithm was used to generate free-shape meta-atoms with controllable feature size and boundary curvature. The spectral response of the unit on the metasurface was enriched with the complexity of Bloch mode. The ultrafast on-chip spectrometer with 356 × 436 pixels composed of a metasurface layer, a microlens layer, and an image sensor layer has been realized, with an average fidelity of 98.78% and a spectral resolution of 0.5 nm.

To acquire spectral images quickly and accurately without complex algorithms, narrow band filtering can obtain spectral information of different wavelengths through a single filter or filter array that can change transmission with time, and its structure is more compact. McClung et al. [66] realized a snapshot spectral camera with a spectral resolution of 7 nm from 795 nm to 980 nm based on a 20-channel parallel narrowband filtered metasurface array (Figure 2f). The structure consists of three cascaded metasurfaces. The metasurfaces on both sides of the first layer of the glass substrate are used to correct monochromatic aberrations. The third layer of metasurfaces deposited on the second substrate is used to filter and correct the waveband aberrations. The filtering range is adjusted by changing the diameter of amorphous silicon atoms. A single shot can simultaneously obtain 20-channel spectral images.

Parameters such as true color gamut, brightness, saturation, resolution, and adjustability in consumer products lead to higher requirements for structural color. Dyeing methods such as pigments and dyes used in traditional industries will be affected by the environment and lack ideal stability and durability. Inspired by the bright colors presented by nanostructures on biological surfaces, scientists have discovered that subwavelength periodic structures can exhibit magnificent colors when they interact with light. By changing the material, arrangement, or structure of the metasurface atoms, the optical resonance can be tuned to produce high-permanence and stable colors. Structural color is mainly realized by localized surface plasmon polariton (SPP) and Mie resonance.

**Figure 3:**
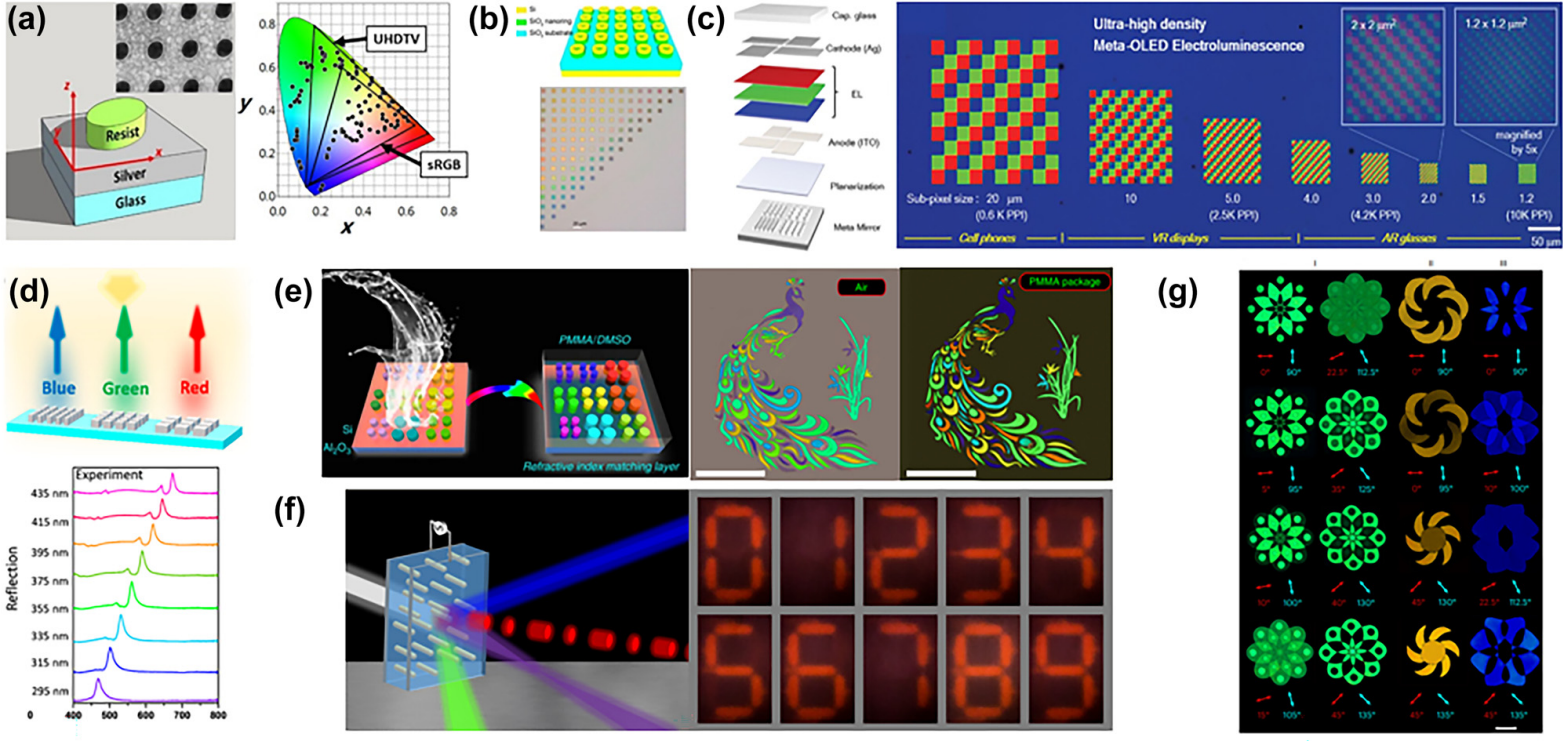
Various structural colors implemented by metasurface. (a) Elliptical dielectric post made of resist ma-N 2401 designed on silver film [70]. (b) Structure diagram of all dielectric metasurface of annular nano unit; bright field optical images of structures with 100 nm atomic spacing [72]. (c) Structure of meta OLED; RGB pixelled ultrahigh density electroluminescence image under conventional optical microscope [73]. (d) The incident light irradiates the red, green, and blue color pixels formed by the silicon nitride super surface; reflectance spectra of 8 structural colors from blue (295 nm) to red (435 nm) of silicon nitride metasurface obtained in the experiment [74]. (e) Adding a refractive index matching layer to reduce the color paleness caused by background reflection in the air; corresponding light field microscope images of peacocks and orchids in the air (left) and packaged with PMMA (right) [75]. (f) The dynamic plasma pixel arrangement is realized by changing the direction of the plasma nanorods in the organic suspension by applying an electric field; the picture of seven segment digital indicators, the contrast of brightness and chroma between the area with applied electric field (bright) and the area without applied electric field (dark), proves the integration prospect of switchable plasma (the outer diameter of “0” is 12.5 × 22 m) [76]. (g) Top view of silver atom embossed on the surface; the plasma steganography technology with kaleidoscope color image switching has experimentally captured images under different polarizer analyzer combinations; encoding the hue and brightness information into the nano aperture with variable geometric size and direction to achieve realistic plasma full-color nano painting [77].

SPP allows strong local confinement of electric fields, which means the nano-engineered structures and incident light can efficiently interact. Based on the uncoupled SPP, Zhao et al. designed circular nanohole-nanodisk hybrid nanostructure arrays [69]. The diameter of the nanostructure affects the resonant wavelength of the uncoupled SPP mode, thereby manipulating the structural color. The minimum pixel shows 141,000 dpi spatial resolution and an angle insensitivity of up to ±40°. Jiang et al. [70] designed a 90 nm high elliptical dielectric post made of resist ma-N 2401 nanostructure on a silver film (Figure 3a), which acts like two dipoles polarized along the major and minor axes of the ellipse and realized circular-polarized reflection geometric phase manipulation. The color gamut is equivalent to approximately 170% of the area of the sRGB color gamut and 98% of the ultrahigh-definition TV (UHDTV) color gamut. FWHM of the cross-polarized reflection spectrum is less than 20 nm. Another work [71] proposed to use of gap plasmon resonances associated with confined aluminum (Al) particle nanocavities on the film to achieve perfect absorption of the incident light, thereby generating reflective complementary colors.

However, the existing mature CMOS processes cannot be compatible with gold, silver, and other materials in the metal nanostructures of plasmons. Due to Ohmic loss, the peak shape will be widened, limiting the color gamut. The dielectric metasurfaces use Mie resonators to realize the resonance of Mie scattering. Different from the plasma metasurface mainly controls the electric dipole; the dielectric meta-atom can effectively control the electric dipole and magnetic dipole due to its high refractive index, providing high-quality factor resonances, which is conducive to the improvement of color gamut. Mie-resonance also has higher-order multipoles, which provide more solutions for the design of structural colors. Since the multi-level mode of the non-resonant wavelength will affect the monochromaticity of the reflection spectrum, which can be solved by using the refractive index matching between SiO_2_, TiO_2_, and Si_3_O_4_ to suppress the multi-level mode excitation in the short wavelength [78]. The refractive index matching between the three dielectric layers successfully suppresses the high-order multilevel modes of short wavelengths, making the reflection spectrum have better monochromatism. An all-dielectric high-refractive index truncated conical metasurface is designed to confine the magnetic Mie resonance field to optimize resonance characteristics and eliminate useless spectral features. The intensity of the reflection peak of the realized structural color is about 90%, and the reflection spectrum of the FWHM is 43 nm. Special shape meta-atoms can manipulate reflection peaks more flexibly. For ring-like atoms, in addition to conventional geometric parameters, the diameter of inner and outer rings can also be adjusted to change the intensity of different resonance modes (Figure 3b) [72]. The incident light interacts with the ring-like atoms to generate electrical and magnetic resonance modes. By adjusting the parameters to change the wavelength of the reflection peak, the achieved structural color covers 115% of the standard color space (sRGB) [79]. However, these structures have poorer field confinement, which is difficult to improve the printing resolutions. To increase the pixel density, vivid colors can also be achieved by employing a Fabry–Perot (FP) cavity. Joo et al. [73] designed a spatially variable hyperphoton FP cavity by combining the metasurface and organic light-emitting diode (OLED) display structure, changing the size of the metasurface nanostructure, changing the reflection phase in the FP cavity, adjusting the resonant frequency, which realized a full-color, high brightness OLED architecture which wavelengths can be adjusted in the visible spectrum. It can increase the pixel density to 10,000 pixels per inch (PPI), meeting the requirements of the next generation of more advanced displays (Figure 3c).

Since the high-order Mie resonance will reduce the saturation of the color, Yang et al. [74] suppress it under the Rayleigh anomaly at short wavelengths. And design a Si3N4 nanoresonator on a quartz substrate to induce magnetic dipole lattice resonance (MDLR) to produce sharper lattice resonances for higher saturation structural colors (Figure 3d). Metasurface can remain bright colors by laterally incident light, and also can achieve pixel switching by changing TE and TM polarization. Due to the high refractive index of Si, there is a high-order mode at the edge band of the reflection spectrum, the color purity is low. Yang et al. [75] designed the refractive index matching layer, which can reduce the refractive index contrast between air and Si (Figure 3e), and push electric dipole resonance to magnetic dipole resonance to obtain narrower reflection spectrum bandwidth. The structural color has a diffraction-limited resolution, and the color gamut covers about 181.8% of sRGB, 135.6% of Adobe RGB, and 97.2% of Rec.2020. Metasurface can also be used to design transmission structure color [80]. AI − SI_3_N_4_ atomic energy deposited on glass substrate introduces magnetic dipole and electric quadrupole resonance in the coupling between Wood anomaly and Mie lattice resonance, thus breaking the symmetric scattering, improving the efficiency of transmission mode, presenting an enhanced resonance peak with FWHM of 50 nm and efficiency of more than 70%.

However, static structural color devices are not convenient in practical applications. How to realize dynamic color display is the research focus of structural color devices. By introducing dynamically adjustable parameters and substances, and adjusting and changing the geometric size, direction, and spacing of structures, dynamic structural color display can be realized. Greybush et al. [76] applied an electric field to the suspension containing nano-element atoms to change the extinction spectrum (Figure 3f), and achieved different structural colors by adjusting the geometric direction of the element atoms. It is also possible to use phase-change materials in crystallization, amorphous and intermediate states to modulate the optical properties of the component surface [81]. In the designed structure, the gap between the Au NPs and the Au mirror has strong electric field confinement. Au NPs are encapsulated in a thin polyaniline (PANI) shell to control the oxidation state. Changes in the refractive index with oxidation state control the scattering cross section, resulting in color changes. Song et al. [77] designed a surface-relief plasmonic metasurface consisting of shallow nanoapertures, which can independently adjust color saturation, hue, brightness, and polarization at the same time (Figure 3g). As a proof of concept, a metasurface artwork of nano painting was made. Besides, the information state of the kaleidoscope was decrypted without crosstalk by adjusting the direction of nanoholes.

Tri-functional metasurface with anisotropic gap-plasmon structures can simultaneously control the phase, amplitude, and light emission. Geometric anisotropy makes the structure show different responses under *x*-polarized light and *y*-polarized light, which display color images under a white light source, and hologram images under a red laser. Besides, by inserting upconversion nanoparticles (UCNPs) into the dielectric gaps of the structures, tuning the size of the gap-plasmon resonators to enhance different emission channels, which can generate different luminescence images [82].

## 3 Polarization-dependent metasurface

Polarization as one of the degrees of freedom (DOFs) of light, contains much information of light, which is of great importance in imaging, displaying and other optical filed. As metasurfaces can adjust the polarization of light pixel by pixel through meta-atoms, using metasurfaces to manipulate the polarization in sub-wavelength scale has become a research hotspot in the field of nano-photonics. By adjusting the size of meta-atoms along the fast and slow axis will impose phase shifts *ϕ*_x_ and *ϕ*_y_ called the propagation phase. Besides, rotating the meta-atoms at an angle *θ* will impose a geometric phase, which will give the opposite phase on arbitrary orthogonal states of polarization. Then, metasurfaces can be described by the Jones matrix [83].

$$J=R\left(-\theta \right)\left[\begin{array}{cc} {\text{e}}^{\text{i}\phi_{x}} & 0\\ 0 & {\text{e}}^{\text{i}\phi_{y}} \end{array}\right]R\left(\theta \right)$$

Here we introduce recent applications of metasurfaces in polarization optics.

**Figure 4:**
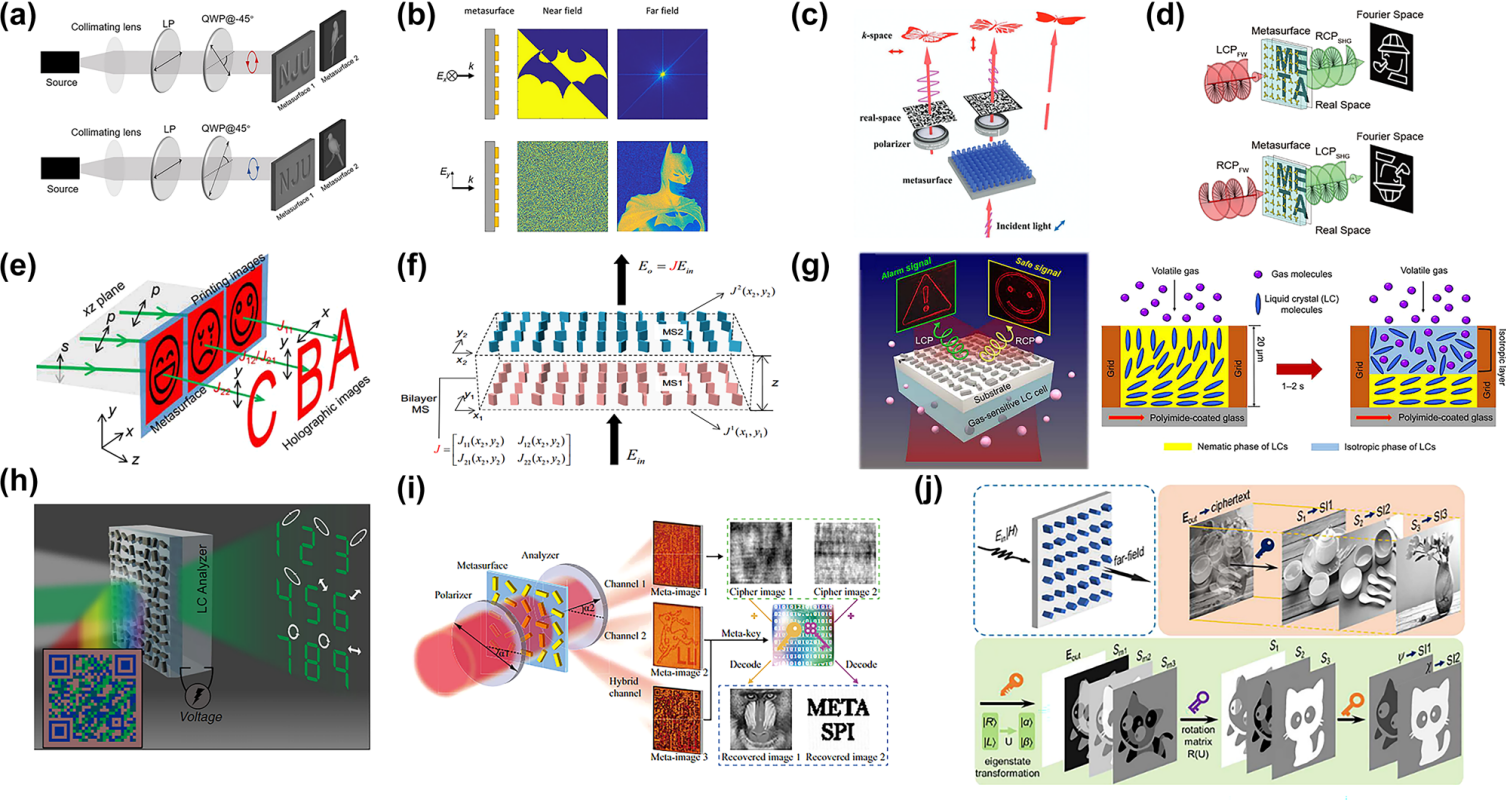
Polarization-dependent hologram and its applications based on metasurface. (a) Two independent nano-printings can be generated under LCP and RCP [87]. (b) Phase imaging can be obtained under *x* polarization and holography under *y* polarization [88]. (c) Metasurface can encode both QR codes in real space and holography in *k*-space [91]. (d) Nonlinear metasurface generates a real space image and holography in the Fourier space [92]. (e) Triple groups of nano-printing and holography can be encoded in a single metasurface with six DOFs Jones matrix under different polarization. And the scanning electron microscopy (SEM) image of the metasurface with four atoms in one pixel [93]. (f) A bilayer metasurface with eight DOFs consists of two metasurfaces with six DOFs [94]. By rotating the second metasurface, it will show different results. (g) A gas sensor integrates a polarization-dependent metasurface and a gas-response LCs, which will output different holography under different gas situations [96]. (h) The system can reflect a QR image with key information and output vectorial holography. By controlling the voltage of the LCs, it will show different secret information [97]. (i) Dual-channel metasurface can show two independent images and a hybrid image, based on which the user can figure out the secret information by using the SPI [98]. (j) The information is hidden in the Stokes vectors. Furthermore, introducing a rotation matrix to the Stokes vector corresponding to the eigenstate transformation to realize asymmetric encryption [99].

By designing anisotropic metaatoms or combinations of anisotropic metasurface units, different amplitude and phase responses can be generated to the polarization state of the input light. Here, a series of works designed polarization-multiplexing metasurfaces. The output of single holograms or nanoprints based on metasurfaces has been investigated [84–86]. Thus, the functionality of polarization-multiplexing metasurfaces is demonstrated through multiple holograms or nanoprinting. Via a couple of staggered meta-atoms as a pixel, the authors realized independent amplitude control of arbitrary orthogonal states of polarization. Two independent nano-printing were generated under left circular polarization (LCP) and right circular polarization (RCP) incidence (Figure 4a) [87]. According to the Jones matrix produced by metasurfaces, two different phase profiles can be added to the *x*-polarization and *y*-polarization light. Based on this, dual-mode metasurfaces were designed to generate near-field phase imaging and far-field holographic under the orthogonal linear polarization. The near-field phase imaging needs a quadriwave lateral shearing interferometry technique to be measured (Figure 4b) [88]. On the one hand, based on Malu’s law, metasurfaces can adjust the amplitude in real space by rotating the angle of the meta-atom [89, 90]. On the other hand, according to the Jones matrix, metasurfaces can control the polarization of transmitted light. Then, under the same polarization of incident light, a metasurface with the ability to generate different gray-scale printings in real space and different holography with different polarization in *k*-space was demonstrated (Figure 4c) [91]. In the nonlinear regime, the second harmonic generation wave will introduce a spin-controlled geometric phase. Moreover, the interference between the wave can modulate the amplitude. A nonlinear plasmonic metasurface is designed, which is composed of gold meta-atoms with C3 rotational symmetry. It achieves real space printing and holography at the same time (Figure 4d) [92]. In order to figure out the upper-limit DOFs of the 2D planar Jones matrix generated by metasurfaces, each pixel of the coherent pixelated metasurface consists of four nano-blocks with different *x*-coordinate positions and orientational angles, which achieves six DOFs. To prove the theory, triple amplitude phase holograms and nano-printing were encoded in three components of the Jones matrix (Figure 4e) [93]. Recently, eight DOFs are realized by a bilayer metasurface that breaks the mirror symmetry of the Jones matrix. Based on this structure, arbitrary phase and amplitude can be imposed on any two polarizations. In addition, by rotating the second metasurface, it can generate 16 holograms under different incident polarization and analyzed polarization (Figure 4f) [94]. By introducing the correlated noise to break the limitation of the Jones matrix and introducing the noncorrelated noise to reduce the cross-talk, the authors realized a polarization-multiplexed metasurface with 11 independent channels [95].

All the metasurfaces above are limited to half-space. Full-space metasurface can work in both reflection and transmission space at the same time and it can manipulate the electromagnetic (EM) wave in full space, which can introduce a new DOF for metasurface [100–102]. Most of the full-space metasurfaces are composed of several layers that need complex fabrication and are hard to integrate. The authors designed a dual-frequency metasurface with a single layer that can control CP wave in full space separately by rotating different metal structures on both sides [103]. The full-space metasurface shows different functions for transmission and reflection modes, which will contribute to realizing the multifunctional metasevices.

The functionality of polarization multiplexing and the compact integration of devices make metasurfaces have significant application value. We will introduce metasurface polarization multiplexing holography in practical scenarios such as environmental detection, information encryption, etc. Liquid crystals (LCs) can be controlled by external stimuli, which can control the output beam polarization by LCs cells. Thus, there are many works integrating LCs with metasurface to make the system tunable [104, 105]. The external stimulation contains electric or magnetic stimulation and so on. Also, when coating the polyimide on the glass substrate, rubbing the polyimide will change the LCs cells to a unidirectional tangential orientation. It is interesting that the volatile gas will lower the LCs ordering, which will change the output polarization. Based on this, integrating the LCs and holographic metasurfaces can produce a gas sensor. As its ultra-compact characteristic, it can be wearable. Thus, it can show different holography under different environments, which can be used in real-time visualization of gas exposure (Figure 4g) [96].

As the light has many DOFs, different combinations of DOFs will increase the information carried by metasurfaces. Only the specified incident light will produce the encrypted information. Therefore, metasurface is a great candidate for optical security [106–110]. Besides, vectorial holography not only controls the amplitude and phase profile but also controls the polarization profile [111]. Combining these two fields, the authors designed an optical security platform consisting of LCs and metasurfaces. On the one hand, two sizes of the meta-atoms are designed to code dual-level grayscale imaging in reflected light, which will tell the user the key. On the other hand, by tailoring the voltage between LCs, the incident polarization will be changed, which will change the output holography. Each super-pixel of the metasurface has nine pixels to generate nine holography. Controlling the proportion of clockwise areas and the phase shift difference between RCP and LCP gives different polarization of the output beams, which achieves vectorial holography (Figure 4h) [97]. So far, most optical security works based on metasurfaces aim to increase the channels of encryption. However, these channels are limited. After multiple attempts, the secret information might be stolen. Combining single-pixel imaging (SPI) with hologram, the secret information will be covered much more securely. The information can be covered into the least significant bit of the holography which can be figured out by specific matrix operations as the keys (Figure 4i) [98]. The authors design four kinds of encryption strategies. First, the secret imaging is hidden in the Stokes vector. After measuring the amplitude of different polarizations, the Stokes vector can be calculated. Furthermore, a polarization mask is selected to introduce a Mueller matrix, which will enhance security. However, the Stokes vector will still expose some information. To solve this issue, the information is hidden in the azimuthal and elliptical angles on the Poincare sphere. Finally, asymmetric encryption is demonstrated, which introduces an eigenstate transformation on the Poincare sphere to change the Stokes vector. Based on this, the keys for encryption and decryption are different (Figure 4j) [99].

**Figure 5:**
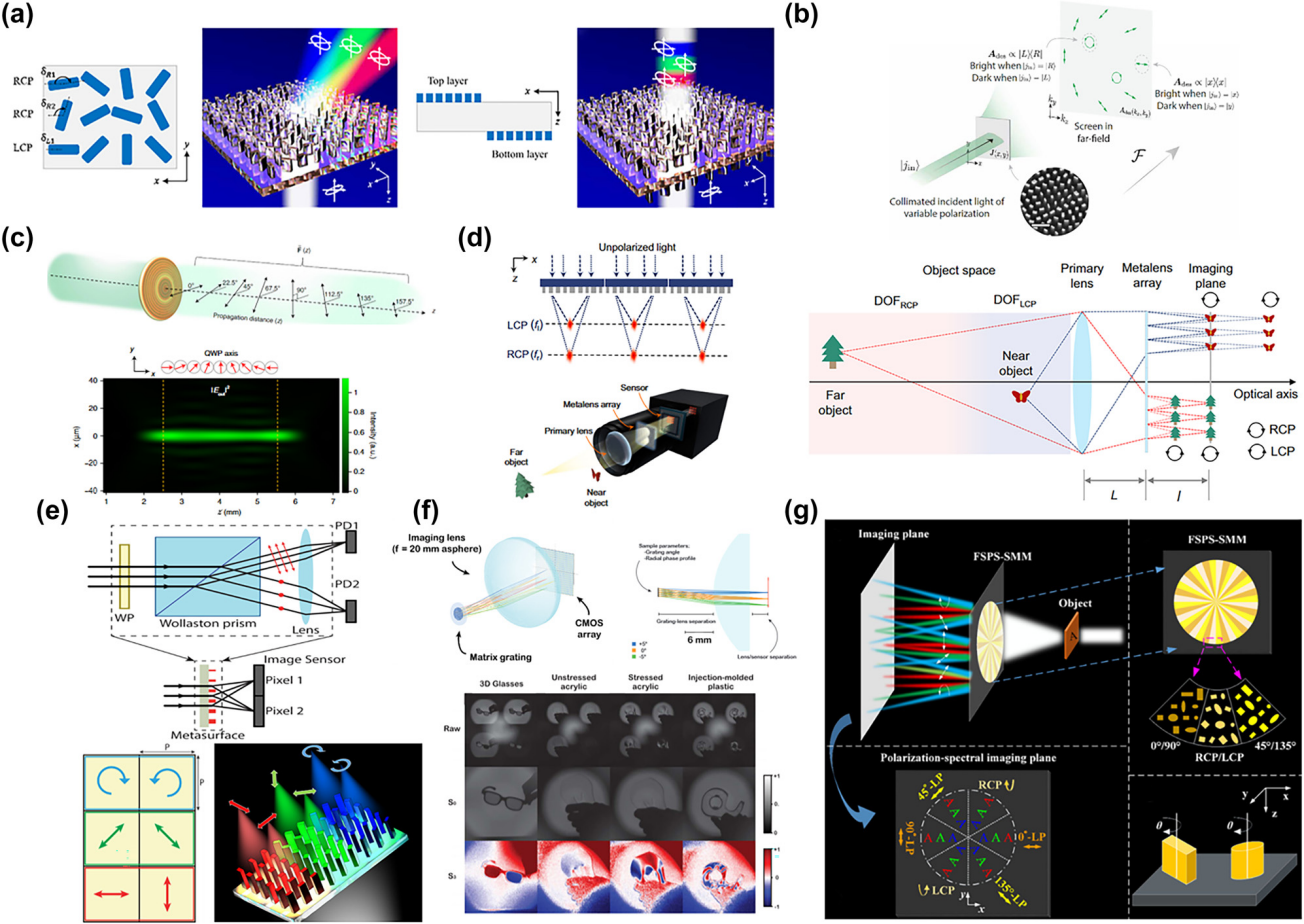
Polarization-dependent metasurfaces used in generating beams with polarization and imaging. (a) Each pixel of the metasurface consists of three lines of meta-atoms with different rotation directions. It realizes broadband polarization-maintain. Adding the bottom layer realizes angular nondispersive at the same time [112]. (b) The metasurface can generate a Jones holography in the far field that can be used in polarization analysis. A waveplate-like metasurface is designed to realize arbitrary linearly birefringent waveplates in different areas [113]. (c) The output polarization rotates along the optical path [114]. (d) The bifocal metalens array will focus LCP and RCP on the near and far points. The near object can be imaged by LCP and the far object can be imaged by RCP at the same time, which realizes the extreme depth of field [115]. (e) Traditional polarization imaging systems can be minimized into a single metasurface. Three couples of polarizations can be focused into different spots to figure out the incident polarization [116]. (f) The metagrating full-Stokes polarization imaging system can image four diffraction orders into different areas and cover 10° FOV [117]. By analyzing the intensity of these four areas, it can get the Stokes vector of the image and figure out the polarization. (g) The spatially multiplexed metasurface can focus different polarization and frequency into various areas to realize a polarization-spectral imaging system [118].

Conventional devices are subject to wavelength-dependent when completing orthogonally polarized output. The authors demonstrated a metasurface with an arrangement of spatially oriented meta-atoms. As the size of all meta-atoms is uniform, it will ignore the wavelength influence. Three lines of phase gradient metasurface have different first rotation angles and gradients, each of which is arranged clockwise or counterclockwise to adjust incident LCP or RCP separately. In the end, the metasurface can generate arbitrary polarization without chromatic dispersion over the entire visible range (Figure 5a) [112]. There is also a demand to achieve different polarization distributions in the transverse plane. Any Jones matrix *J* can be broken into a Hermitian and a unitary matrix, which can be shown as *J* = HU. U can be regarded as the phase term and H can be regarded as the amplitude term. By adjusting the traditional GS algorithm to the matrix GS algorithm, the polarization distribution in the far fields will be controlled, which can show a Jones holographic. This can be used as a visual full-Stokes analyzer. Based on Malus’s law, any incident polarization can be figured out by reading out the amplitude of different areas in the holographic. Besides, this work designs a waveplate, whose phase delay varies from 0 to *π* along the radius, and the orientation of the fast-axis changes with rotation. Finally, different areas of the output polarization will be specific (Figure 5b) [113]. Additionally, there is a new method to realize the polarization shift along the propagation direction. Based on spatial polarization beating and dual matrix holography, the polarization along the optical path rotates independently of the incident polarization (Figure 5c) [114].

Polarization is an important information dimension in imaging, which reveals features that can’t be seen with traditional cameras. Light-field imaging can get the 4D information of the light. In recent years, light-field imaging based on metalens array has attracted much attention, aiming to explore large depth-of-field and high spatial resolution [50, 62]. However, these two features are coupled, making it hard to realize both at the same time. A polarization-multiplexed metalens array is introduced to break the constraint, which provides two focus lengths under a pair of orthogonal circular polarization incidences. Combining the bifocal metalens and instant chromatic aberration, the far boundary of depth-of-field under LCP and the near boundary of the depth-of-field under RCP are connected. Therefore, it will generate a constant point spread function (PSF), which carries the depth information of the object. Finally, the neural network can figure out the depth information of the PSF and eliminate the aberrations (Figure 5d) [115]. Besides, full-stokes imaging measures the polarization information of the object, which can show the texture of the surface, surface stress, and other information that cannot be seen directly. Polarization imaging is widely used in astronomy, biomedical, and so on [119, 120]. A metasurface is designed to split any two orthogonal states of the polarization and focuses them on different areas. The metasurface consists of three areas. They can separately divide horizontal/vertical, antidiagonal/diagonal, and RCP/LCP into different points. Based on the amplitude of these six images, arbitrary polarization can be calculated through the Stokes parameters (Figure 5e) [116]. The light path diagram is shown in (Figure 5f) with a matrix grating that will lead four polarization states into four diffraction orders with high efficiency following an aspheric lens and a CMOS sensor to get the image. The system can cover a 10° FOV finally it successfully supports a snapshot, compact full-Stokes polarization imaging [117]. In addition, the authors design three sub-metalens to demonstrate a metasurface consisting of 12 spatial areas. Different polarization and frequency lights will focus on various points. Apart from full-Stokes imaging, the spectrum of the object can be obtained by lateral chromatic aberration. As a result, they design a full-Stokes polarization-spectral imaging system (Figure 5g) [118].

## 4 Metasurface orbital angular momentum manipulation

Increasing the capability of optical manipulated multiplexing channels is a great challenge, and the emerging field of nanophotonics has unprecedented control over the properties of light at the nanoscale. These challenges are expected to be overcome by utilizing optical angular momentum, the OAM as an information carrier that can meet the growing demands of high-capacity optical information devices. Metasurfaces complete new work in the field of information optics, leading the development of next-generation ultra-high-speed, ultra-high-capacity, and miniaturized information technology devices.

**Figure 6:**
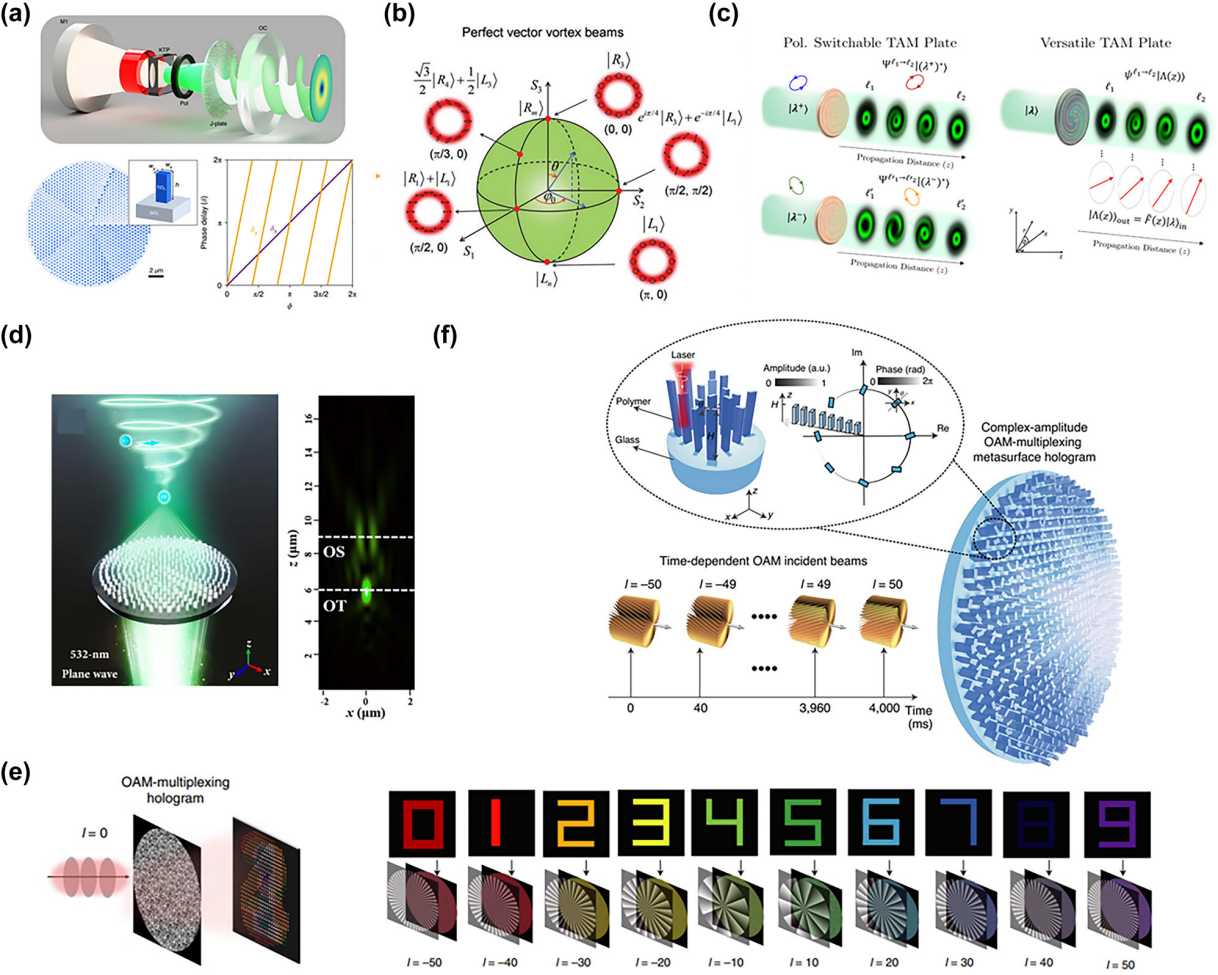
Metasurface manipulates angular momentum. (a) (Top) Illustration of laser cavity with an intracavity nonlinear crystal (KTP), polarizer (Pol), and metasurface (J-plate). (Bottom) Schematic diagram of the central part of the metasurface, the desired phase delay profile for different output OAM [122]. (b) Schematic illustration of the generation of perfect vector vortex beams with arbitrary phase and polarization in a mixed-order Poincaré sphere and under amplitude control with a fixed annular intensity distribution [123]. (c) (Left) Schematic of the proposed polarization-controlled TAM plate (right) schematic of a multipurpose TAM plate that can control the distribution of polarization and orbital angular momentum along the optical path [124]. (d) (Left) Schematic diagram of the polarization-dependent OT–OS metasurface. (Right) Axial cross-sectional view of the intensity distribution. Dashed lines indicate the focal planes of OT and OS functions [125]. (e) (Left) Schematic illustration of an OAM multiplexed hologram. (Right) for a 10 bit OAM-multiplexing hologram [126]. (f) Schematic diagram of the principle of large-scale complex-amplitude OAM-multiplexing metasurface hologram [127].

By arranging V-shaped nano-antenna resonators on a plane, a helical phase-generating OAM beam can be demonstrated by metasurface [23]. Based on the principle of geometric phase, there is a direct link between spin angular momentum and orbital angular momentum generation. LCP and RCP light generate vortex beams with completely opposite topological charges. However, the OAM generated under the geometric phase is dependent, which limits the diversity of the channels. Devlin et al. broke through the limitation of using a single geometric phase by designing a metasurface consisting of the dynamic phase and geometric phase. Left and right circular polarizations can output states with independent OAM values, and further, provide a transition from any orthogonal polarization to completely independent OAM states. Metasurface design is based on breaking the conjugate symmetry between circular spin and OAM states [121]. OAM lasing modes that simultaneously emit independently distributed topological charges with a large gap can be realized. This is an extreme violation of previous symmetric spin–orbit laser devices, and such OAM lasers can provide compact source solutions for applications ranging from imaging to communication (Figure 6a) [122].

In higher dimensions, the goal is to realize the simultaneous regulation of spin angular momentum and orbital angular momentum. Bao et al. [123] proposed to independently and arbitrarily control the amplitude, phase, and polarization of light by using the position and rotation angle of two identical crystalline silicon nanopillars as degrees of freedom for geometric parameters. In this paper, a perfect vector vortex beam with arbitrary polarization and phase distribution is successfully generated. And a constant intensity distribution independent of the topological charge and polarization order is achieved (Figure 6b). Dorrah et al. have further realized the structural distribution of spin angular momentum and orbital angular momentum along the propagation direction, which can potentially extend such applications to 3D. By introducing an arbitrary pair of orthogonal polarizations coupled to the phase planes of two vortices where the magnitude of the vorticity varies locally with propagation and a multifunctional plate that can independently construct the two angular momentums of spin and orbit (Figure 6c) [124]. In addition, Liu et al. [128] demonstrated a novel liquid crystal geometric phase optical element, volumetric OAM that switches between different states on a high-order Poincaré sphere by changing the incident spin, to achieve on-demand OAM beam 3D cropping.

Optical tweezers (OT) and optical spanners (OS) are powerful tools for optical manipulation, which are responsible for particle trapping and rotation, respectively. In 2021, Li et al. [125] completed a metasurface-based solution to integrate OT and OS. Using mainstream methods based the on geometric phase and dynamic phase, an output field with a high numerical aperture focal spot accompanied by coaxial eddy currents can be constructed (Figure 6d).

Encoding OAM information into a holographic phase plate to achieve channel multiplexing, this complex beam with special amplitude and phase distribution has been proven to play an important role in the field of digital holography. Due to the orthogonality of different topological charges of OAM, the image can be successfully decoded in the far field only when the correct OAM beam passes through the structured light-dependent CGH. Ren et al. [129] demonstrated the ability to reconstruct a four-channel OAM multiplexed hologram from a single metahologram via strong OAM selectivity under metasurface design. Further Fang et al. demonstrated OAM holography by finding strong OAM selectivity in the spatial frequency domain without theoretical helical mode refractive index confinement. Its helical pattern has a helical pattern index from 50 to 50, resulting in a 10 bit OAM-encoded hologram for high-security optical encryption (Figure 6e) [126]. Not limited to phase encoding, Ren et al. achieve holography by engineering complex amplitude metasurface in momentum space. The surface is designed to achieve independent amplitude and phase manipulation through the angle of rotation and height. Here we demonstrate an OAM holography technique capable of multiplexing up to 200 independent OAM channels, using different OAM modes of light to extract information via Fourier transform, enabling lens-free reconstruction and holographic video display (Figure 6f) [127].

## 5 Angle-dependent metasurface

**Figure 7:**
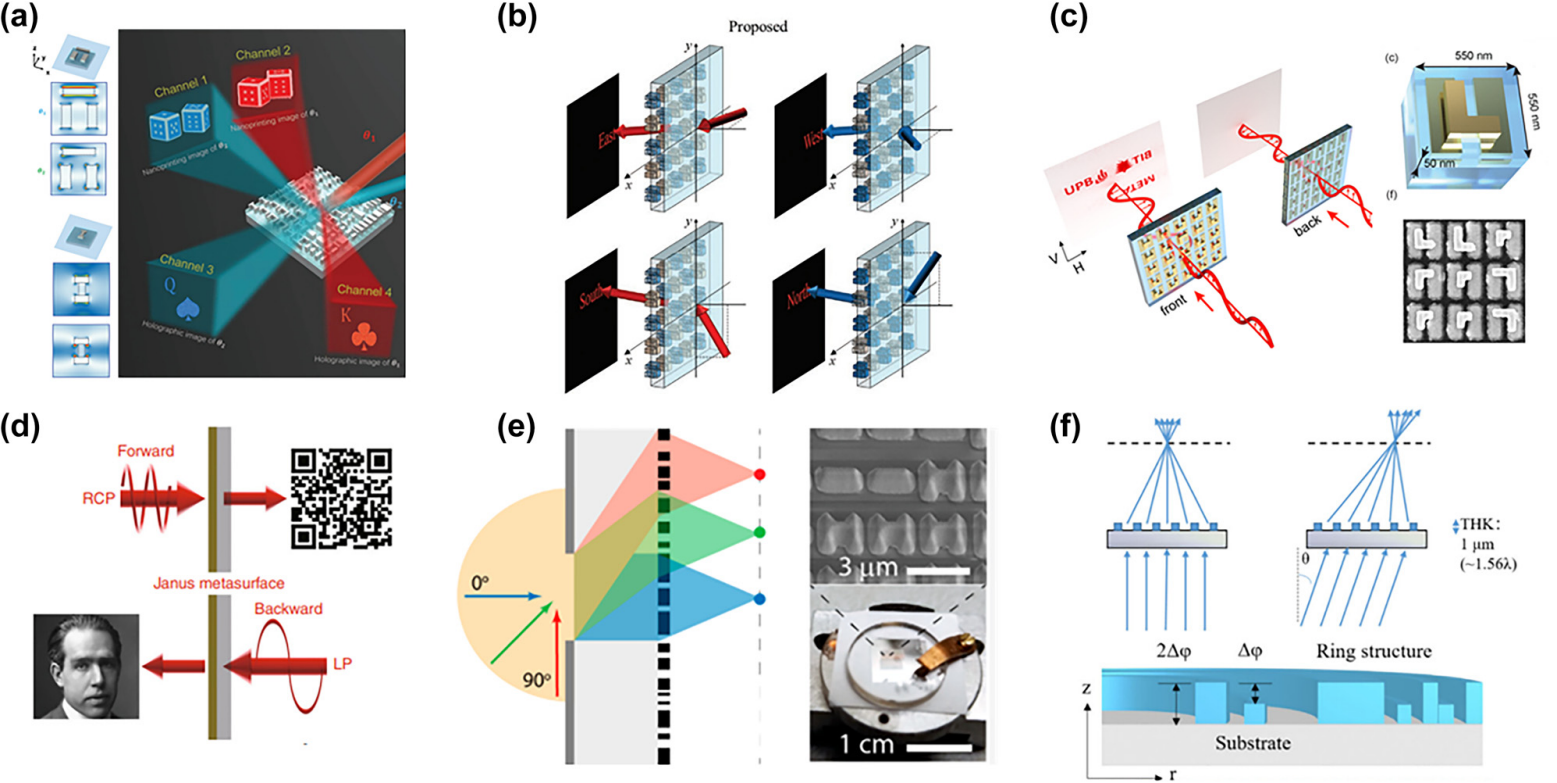
Angle multiplexing manipulation based on metasurface (a) Coupled metal–insulator–metal interatomic geometry in which the electric energy density distribution depends on the illumination angle. Different lighting angles cause independent-encoded nanoprinting images (channels 1 and 2) and holographic images (channels 3 and 4) [133]. (b) Schematic diagram of the function of the detour phase multiplexing hologram [134]. (c) (Left) Schematic of the nonreciprocal functionality of two-layer polarization-sensitive metasurface holograms. Front illumination reveals the hologram, while back illumination hides it. (Right) Double-layer metasurface structure for nonreciprocal holography and corresponding scanning electron microscope image of the double-layer structure [135]. (d) Direction-controlled polarization-encrypted data storage demonstration using Janus metasurfaces. Forward circular polarization output two-dimensional code, and backward linear polarization output Niels Bohr’s photograph [136]. (e) (Left) Schematic diagram of a single-layer metalens over a near 180° angular FOV. (Right) Optical image and SEM image of single-element fisheye metalens [137]. (f) (Top) Schematic diagram of SLAC planar lens focusing on-axis and off-axis light (bottom) schematic diagram of nanostructure of SLAC planar lens [138].

In many practical applications, we require optics to be tolerant to the angle of incidence, and in traditional lensing and beam steering applications, the angular dependence often appears in the form of diffraction losses, distortion, or coma aberration. This requirement has motivated researchers to study metasurfaces with strong angle insensitivity to address these issues. On the other hand, angle sensitivity can also be used to realize new functions of angle functions. Independent control of the response from different angles of incidence can also be well exploited in angular degrees of freedom through interactions between neighboring meta-atoms, often using sharp resonances to jointly modify the output in momentum space. The resonance of the metasurface unit enables multi-angle multiplexing to generate decoupled holographic images under different incident angles. The dielectric U-shaped meta-atoms act as multimode resonators, producing independently controlled angular responses under different angles of illumination [130]. Achieving desired angular dispersion by controlling the near-field coupling between meta-atoms and the radiation pattern of individual meta-atoms can also bring about phase control at different angles [131]. The geometric phase at different angles and rotations can bring considerable degrees of freedom for discrete angle control [132]. Under the resonant coupling between atoms, different phase and amplitude modulations under angle multiplexing of the metasurface can be realized. The extension of the intensity degrees of freedom enables any combination of optical responses with different angles of illumination to enable four-channel output of holographic and amplitude patterns (Figure 7a) [133]. Combining detoured phase holograms with a spatial multiplexing approach allows four phase distributions to be recorded in a single metasurface device. Therefore, holograms with different responses can be obtained under four different incident angles (Figure 7b) [134].

The response depending on the direction of incident light propagation is called a Janus metasurface. This is taken from the Roman Janus, where the two heads looking to the sides represent the past and the future respectively. The concept of a dual-mode metasurface is proposed to control the phase and spectral response of both transmission and reflection modes of operation. In the transmission mode, the dual-mode metasurface acts as a regular metasurface by adjusting the phase distribution of the incident light. In reflective mode, white light illumination produces a reflected color image [139]. Frese et al. introduces a locally asymmetric two-layer metal metasurface design. The design yields bidirectional functionality with full phase and amplitude control of the transmitted light. The coded hologram is designed to appear in a specific linear cross-polarization channel and disappear in the counter-propagation direction (Figure 7c) [135]. A unique type of 3D Janus metallic helical nanoapertures was reported. By encoding the Janus metasurface with two nanoaperture antipodes with a specific rotation angle, the image is displayed in the forward direction under circularly polarized incidence at a specified hand, and in the reverse direction under linearly polarized illumination with a specified azimuthal angle different grayscale images (Figure 7d) [136].

Additionally, Chen et al. [140] demonstrated a passive Janus metasurface composed of cascaded subwavelength anisotropic impedance sheets. By introducing a rotational twist in their geometry, asymmetric transport with the desired phase function is achieved. Kim et al. [141] discovered a novel and exotic optical phenomenon involving tunable color switches for transmitting viewing-related information. Chu et al. [142] introduced a randomly flipped hypersurface composed of randomly flipped components to achieve diffuse reflection on the front and distortion-free transmission on the back.

High-quality imaging requires dealing with conflicts between lens parameters (the trade-off between NA and FOV to correct for multiple aberrations). Metalenses are competitors to replace traditional optical elements in integrated optics or microscale optics. Metalens can achieve monochromatic aberration correction and dispersion engineering functions well through the multifunctional manipulation ability of electromagnetic fields. Despite the remarkable performance, multiple theoretical and experimental challenges must be overcome to continue to develop metasurface-based optical applications. How to replace bulky and complex traditional lenses with metasurfaces has recently been completed.

Extending FOV by double-layer metasurface has been demonstrated [143, 144]. The required number of metasurface layers can be reduced by designing the aperture. In (Figure 7e), the large-angle fisheye lens can be realized by combining the aperture with a single-layer metasurface, and the maximum theoretically can complete 180° FOV. The lens corrects third-order Seidel aberrations including coma, astigmatism, and field curvature [137]. However, the single-layer metasurface design is printed on the other side of the substrate due to the aperture, the thickness is not small. The most ideal state is natural to achieve high-quality large-angle imaging in the case of a single-layer metasurface. Based on the *ɛ*-greedy algorithm scheme, a planar-wavelength-thick single-layer aberration-compensated (SLAC) planar lens composed of a 3D-printed dielectric nanoring structure is proposed. Through optimization, the single-layer metasurface can realize an ultra-thin wide-field-of-view planar lens design (Figure 7f) [138]. Catenary optics is also a design to achieve single-layer metasurfaces. Nearly 100% maximum diffraction efficiency is obtained over an ultra-wide spectral and angular range through a novel equiphase streamline optimization method using a true local geometric phase [145].

**Figure 8:**
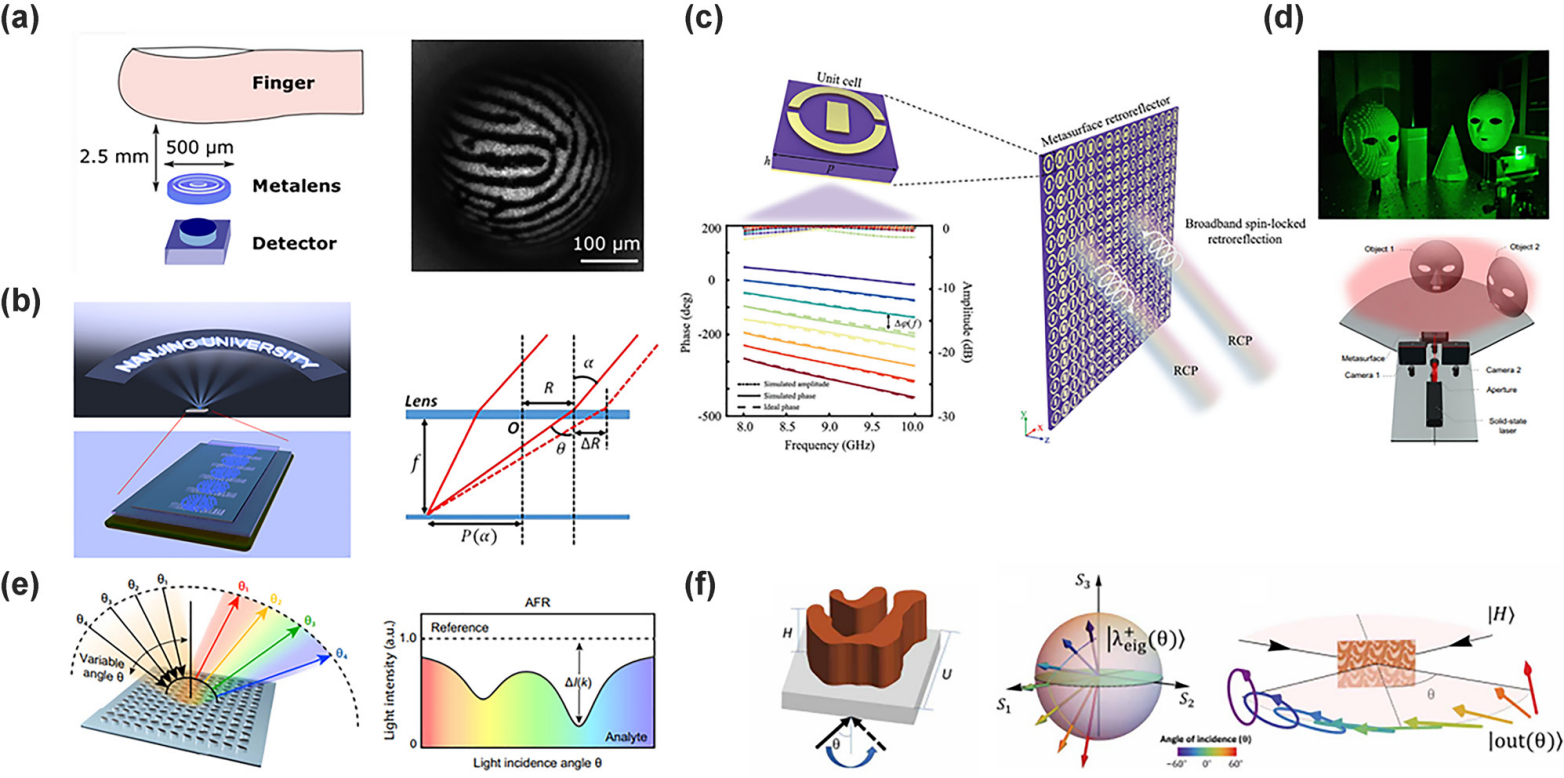
Functional multi-angle metasurface imaging and high-dimensional control (a) (Left) The metalens based on the quadratic phase distribution complete the imaging experiment with 100 fingerprint features (right) experimental images of the fingerprint taken with metalens [148]. (b) (Left) Schematic diagram of wide-angle camera imaging. The magnification shows that each part of the wide-angle image is sharply imaged by the individual metalens. (Right) Schematic design of metalens focusing oblique incident light path [149]. (c) Design and schematic diagram of a broadband spin-locked metasurface retroreflector. The top inset shows the unit cell. The bottom inset shows the reflection amplitude (dashed line) and phase response (solid line) of the eight cells that make up the entire metasurface [151]. (d) (Top) Metasurface-based SL imaging platform scatters a high-density diffracted beam into the entire 180° FOV (bottom) schematic of the optical setup for depth estimation using a 2D array of points scattered on an object [152]. (e) (Left) Schematic diagram of the optimized cell structure (middle) different arrows indicate intrinsic polarization states at different incident angles (right) [153]. (f) Schematic diagram of device operation. As the angle of incidence changes, for a fixed polarization of the incident ray, the outgoing ray changes from right circular polarization to horizontal linear polarization to 45° linear polarization [154].

Fourier lens is a method that can realize large-angle imaging of single-layer lenses. By operating in a single layer, optical coma aberration at large angles can be controlled. Liu et al. [146] realize a dielectric metasurface consisting of an array of high-aspect-ratio silicon waveguides capable of performing a one-dimensional Fourier transform over a wide range of incidence angles. Martins et al. [147] demonstrated the ability of single-layer metallic films to perform wide FOV imaging while maintaining a high resolution suitable for most applications by relaxing the constraints of diffraction-limited resolution. Lassalle et al. [148] analyzed the imaging characteristics of quadratic phase lenses, the achievable FOV for a given imaging configuration, and the optical resolution. To illustrate the full potential of quadratic metalens, complete imaging of a 5 mm fingerprint with features on the order of 100 μm is experimentally demonstrated, with the metalens only 2.5 mm from the object (Figure 8a). Chen et al. [149] designed metalens array that intentionally introduces a phase-shifting term, realizes imaging at different angles, and finally achieves large-angle aiming. After the stitching process, they obtained images with a large viewing angle of >120°, and the entire lens array can capture scenes with large viewing angles and negligible distortion or aberration (Figure 8b). Li et al. [150] proposed a new framework for establishing boundaries, positing that there is an inherently constrained trade-off between achieving the desired wide-angle response and reducing device thickness.

In addition to the continuous angle processing of imaging, the retroreflective optical operation of continuous angle light returning along the original path is also an important application field for metasurfaces. Arbabi et al. [155] through the double-layer metasurface, one layer realizes the Fourier optical operation, the other layer realizes the transverse gradient phase, and the retroreflection effect can be realized. Through the resonator effect, the single-layer ultra-thin metasurface can realize retroreflection. Tan et al. [151] design low-loss dielectric resonators and metasurface reflectors that can achieve polarization independence and dispersion compensation by introducing the propagation phase and geometric phase. The proposed metasurface can achieve retroreflection over a broadband spectrum while keeping the spin state unchanged (Figure 8c). Metasurfaces can also generate multi-angle outputs for a single incident response. Through metasurface phase design, multi-point output effects can be achieved. This plays an important role in 3D facial recognition, Lidar space recognition, etc. Rho et al. proposed a metasurface-enhanced SL-based depth-sensing platform that scatters a high-density array of 10 K points across a 180° FOV by manipulating light on a subwavelength scale (Figure 8d) [152]. Martins et al. [156] reported an advanced lidar technique. The technique utilizes an ultrafast low FOV deflector cascaded with a metasurface to achieve a large FOV (150°) and a high frame rate (kHz), providing both peripheral and central imaging area. Based on the quasi-BIC, Leitis et al. [153] realized broadband spectral absorption for different incident angles, and completed broadband spectral control by using the degree of freedom of the angle (Figure 8e). In addition, Shi et al. [154] demonstrated the use of topology-optimized metasurface structures. The incident angle function is continuously tuned from linear birefringence to elliptical birefringence through the device behavior, enabling compact and versatile polarization manipulation (Figure 8f).

## 6 Conclusions and outlook

In recent years, metasurface has been used in various optical fields owing to its ability to control DOFs of light at the subwavelength scale. We have reviewed recent applications of metasurface in imaging, display, structured light control, and other optical fields based on controlling the wavelength, polarization, OAM, and angle of the incident light. Metasurface can combine several DOFs to increase the complexity of information-encoding, such as polarization and OAM multiplexing [95], polarization and wavelength multiplexing [157], and so on [153, 154]. With the development of metasurfaces, complex manipulation of more dimensions can be integrated into two-dimensional planes, which greatly improves the compactness of optical devices.

Due to the compact size, metasurface can be wildly used in augmented reality (AR) [158–160], virtual reality (VR) [161], and light detecting and ranging (LIDAR) [156, 158, 162, 163]. To explore more capabilities, the bilayer metasurface is introduced to explore the capabilities of greater manipulation, that can’t be generated by the single metasurface. Inverse design based on optimization algorithms or machine learning is also introduced. Inverse design helps us achieve unique metasurface performance, especially those problems that cannot be solved intuitively by traditional design methods. How to develop the inverse design algorithm fast and precisely remains the research hotspot [164, 165]. However, traditional metasurface is passive. A dynamic and compact optical element is appealing. By integrating the metasurface with LCs, digital micromirror devices (DMD), spatial light modulators (SLM), or other dynamic devices [166]. To further minimize the size of the system, phase-change materials are introduced. They can change their optical response under electrical, thermal, mechanical, and light stimulation [167, 168]. The spatiotemporal metasurface can achieve more capabilities. How to further increase the refresh speed and information capability are the research trends. In order to deliver a commercial application, the fabrication of the metasurface needs to be more precise and inexpensive.

## References

[1] Cheben P., Halir R., Schmid J. H., Atwater H. A., Smith D. R. (2018). Subwavelength integrated photonics. Nature.

[2] Davoyan A., Atwater H. (2018). Quantum nonlinear light emission in metamaterials: broadband purcell enhancement of parametric downconversion. Optica.

[3] Dyachenko P. N., Molesky S., Petrov A. Y. (2016). Controlling thermal emission with refractory epsilon-near-zero metamaterials via topological transitions. Nat. Commun..

[4] Huang Y. W., Lee H. W. H., Sokhoyan R. (2016). Gate-tunable conducting oxide metasurfaces. Nano Lett..

[5] Jahani S., Jacob Z. (2016). All-dielectric metamaterials. Nat. Nanotechnol..

[6] Jahani S., Kim S., Atkinson J. (2018). Controlling evanescent waves using silicon photonic all-dielectric metamaterials for dense integration. Nat. Commun..

[7] Kapitanova P. V., Ginzburg P., Rodríguez-Fortuño F. J. (2014). Photonic spin hall effect in hyperbolic metamaterials for polarization-controlled routing of subwavelength modes. Nat. Commun..

[8] Krishnamoorthy H. N., Jacob Z., Narimanov E., Kretzschmar I., Menon V. M. (2012). Topological transitions in metamaterials. Science.

[9] Kuznetsov A. I., Miroshnichenko A. E., Brongersma M. L., Kivshar Y. S., Luk’yanchuk B. (2016). Optically resonant dielectric nanostructures. Science.

[10] Liu Z., Zhu D., Rodrigues S. P., Lee K. T., Cai W. (2018). Generative model for the inverse design of metasurfaces. Nano Lett..

[11] Nicholls L. H., Rodríguez-Fortuño F. J., Nasir M. E. (2017). Ultrafast synthesis and switching of light polarization in nonlinear anisotropic metamaterials. Nat. Photonics.

[12] Park Y., Depeursinge C., Popescu G. (2018). Quantitative phase imaging in biomedicine. Nat. Photonics.

[13] Rho J., Ye Z., Xiong Y. (2010). Spherical hyperlens for two-dimensional sub-diffractional imaging at visible frequencies. Nat. Commun..

[14] Sherrott M. C., Hon P. W., Fountaine K. T. (2017). Experimental demonstration of > 230 phase modulation in gate-tunable graphene–gold reconfigurable mid-infrared metasurfaces. Nano Lett..

[15] Shi Y., Li H., Li L. J. (2015). Recent advances in controlled synthesis of two-dimensional transition metal dichalcogenides via vapour deposition techniques. Chem. Soc. Rev..

[16] Thyagarajan K., Sokhoyan R., Zornberg L., Atwater H. A. (2017). Millivolt modulation of plasmonic metasurface optical response via ionic conductance. Adv. Mater..

[17] Wang C., Zhang M., Chen X. (2018). Integrated lithium niobate electro-optic modulators operating at cmos-compatible voltages. Nature.

[18] Wen D., Yue F., Li G. (2015). Helicity multiplexed broadband metasurface holograms. Nat. Commun..

[19] Zhang L., Chen X. Q., Liu S. (2018). Space-time-coding digital metasurfaces. Nat. Commun..

[20] Murray W. A., Barnes W. L. (2007). Plasmonic materials. Adv. Mater..

[21] Ni X., Wong Z. J., Mrejen M., Wang Y., Zhang X. (2015). An ultrathin invisibility skin cloak for visible light. Science.

[22] Sun S., Yang K. Y., Wang C. M. (2012). High-efficiency broadband anomalous reflection by gradient meta-surfaces. Nano Lett..

[23] Yu N., Genevet P., Kats M. A. (2011). Light propagation with phase discontinuities: generalized laws of reflection and refraction. Science.

[24] Mahmood N., Kim I., Mehmood M. Q. (2018). Polarisation insensitive multifunctional metasurfaces based on all-dielectric nanowaveguides. Nanoscale.

[25] Berry M. V. (1987). The adiabatic phase and pancharatnam’s phase for polarized light. J. Mod. Opt..

[26] Chen X., Huang L., Mühlenbernd H. (2012). Dual-polarity plasmonic metalens for visible light. Nat. Commun..

[27] Dai J. Y., Zhao J., Cheng Q., Cui T. J. (2018). Independent control of harmonic amplitudes and phases via a time-domain digital coding metasurface. Light: Sci. Appl..

[28] Liu L., Zhang X., Kenney M. (2014). Broadband metasurfaces with simultaneous control of phase and amplitude. Adv. Mater..

[29] Wang L., Kruk S., Tang H. (2016). Grayscale transparent metasurface holograms. Optica.

[30] Chen X., Zhang Y., Huang L., Zhang S. (2014). Ultrathin metasurface laser beam shaper. Adv. Opt. Mater..

[31] Khorasaninejad M., Chen W. T., Devlin R. C., Oh J., Zhu A. Y., Capasso F. (2016). Metalenses at visible wavelengths: diffraction-limited focusing and subwavelength resolution imaging. Science.

[32] Pu M., Li X., Ma X. (2015). Catenary optics for achromatic generation of perfect optical angular momentum. Sci. Adv..

[33] Wu K., Coquet P., Wang Q. J., Genevet P. (2018). Modelling of free-form conformal metasurfaces. Nat. Commun..

[34] Huang Y. W., Chen W. T., Tsai W. Y. (2015). Aluminum plasmonic multicolor meta-hologram. Nano Lett..

[35] Arbabi A., Horie Y., Bagheri M., Faraon A. (2015). Dielectric metasurfaces for complete control of phase and polarization with subwavelength spatial resolution and high transmission. Nat. Nanotechnol..

[36] Chen W. T., Török P., Foreman M. R. (2016). Integrated plasmonic metasurfaces for spectropolarimetry. Nanotechnology.

[37] Khorasaninejad M., Chen W., Zhu A. (2016). Multispectral chiral imaging with a metalens. Nano Lett..

[38] Chen W. T., Zhu A. Y., Capasso F. (2020). Flat optics with dispersion-engineered metasurfaces. Nat. Rev. Mater..

[39] Meng Y., Chen Y., Lu L. (2021). Optical meta-waveguides for integrated photonics and beyond. Light: Sci. Appl..

[40] Qin J., Jiang S., Wang Z. (2022). Metasurface micro/nano-optical sensors: principles and applications. ACS Nano.

[41] Fu R., Chen K., Li Z., Yu S., Zheng G. (2022). Metasurface-based nanoprinting: principle, design and advances. *Opto-Electron. Sci.*.

[42] Kim J., Seong J., Yang Y., Moon S. W., Badloe T., Rho J. (2022). Tunable metasurfaces towards versatile metalenses and metaholograms: a review. Adv. Photonics.

[43] Hsu W. L., Chen Y. C., Yeh S. P., Zeng Q. C., Huang Y. W., Wang C. M. (2022). Review of metasurfaces and metadevices: advantages of different materials and fabrications. Nanomaterials.

[44] Aieta F., Kats M. A., Genevet P., Capasso F. (2015). Multiwavelength achromatic metasurfaces by dispersive phase compensation. Science.

[45] Khorasaninejad M., Aieta F., Kanhaiya P. (2015). Achromatic metasurface lens at telecommunication wavelengths. Nano Lett..

[46] Khorasaninejad M., Shi Z., Zhu A. Y. (2017). Achromatic metalens over 60 nm bandwidth in the visible and metalens with reverse chromatic dispersion. Nano Lett..

[47] Wang S., Wu P. C., Su V. C. (2017). Broadband achromatic optical metasurface devices. Nat. Commun..

[48] Wang S., Wu P. C., Su V. C. (2018). A broadband achromatic metalens in the visible. Nat. Nanotechnol..

[49] Chen W. T., Zhu A. Y., Sanjeev V. (2018). A broadband achromatic metalens for focusing and imaging in the visible. Nat. Nanotechnol..

[50] Lin R. J., Su V. C., Wang S. (2019). Achromatic metalens array for full-colour light-field imaging. Nat. Nanotechnol..

[51] Fan Z. B., Qiu H. Y., Zhang H. L. (2019). A broadband achromatic metalens array for integral imaging in the visible. Light: Sci. Appl..

[52] Shrestha S., Overvig A. C., Lu M., Stein A., Yu N. (2018). Broadband achromatic dielectric metalenses. Light: Sci. Appl..

[53] Chen W. T., Zhu A. Y., Sisler J., Bharwani Z., Capasso F. (2019). A broadband achromatic polarization-insensitive metalens consisting of anisotropic nanostructures. Nat. Commun..

[54] Wang Y., Chen Q., Yang W. (2021). High-efficiency broadband achromatic metalens for near-ir biological imaging window. Nat. Commun..

[55] Xiao X., Zhao Y., Ye X. (2022). Large-scale achromatic flat lens by light frequency-domain coherence optimization. Light: Sci. Appl..

[56] Chen C., Song W., Chen J. W. (2019). Spectral tomographic imaging with aplanatic metalens. Light: Sci. Appl..

[57] Sisler J., Chen W. T., Zhu A. Y., Capasso F. (2020). Controlling dispersion in multifunctional metasurfaces. APL Photonics.

[58] Zou X., Zhang Y., Lin R. (2022). Pixel-level bayer-type colour router based on metasurfaces. Nat. Commun..

[59] Miyata M., Nemoto N., Shikama K., Kobayashi F., Hashimoto T. (2021). Full-color-sorting metalenses for high-sensitivity image sensors. Optica.

[60] Faraji-Dana M., Arbabi E., Kwon H. (2019). Hyperspectral imager with folded metasurface optics. ACS Photonics.

[61] Billuart J., Héron S., Loiseaux B., Amra C., Lequime M. (2021). Towards a metasurface adapted to hyperspectral imaging applications: from subwavelength design to definition of optical properties. Opt. Express.

[62] Hua X., Wang Y., Wang S. (2022). Ultra-compact snapshot spectral light-field imaging. Nat. Commun..

[63] Wang Z., Yi S., Chen A. (2019). Single-shot on-chip spectral sensors based on photonic crystal slabs. Nat. Commun..

[64] Xiong J., Cai X., Cui K. (2022). Dynamic brain spectrum acquired by a real-time ultraspectral imaging chip with reconfigurable metasurfaces. Optica.

[65] Yang J., Cui K., Cai X. (2022). Ultraspectral imaging based on metasurfaces with freeform shaped meta-atoms. Laser Photonics Rev..

[66] McClung A., Samudrala S., Torfeh M., Mansouree M., Arbabi A. (2020). Snapshot spectral imaging with parallel metasystems. Sci. Adv..

[67] Lee J., Park Y., Kim H. (2022). Compact meta-spectral image sensor for mobile applications. Nanophotonics.

[68] Wu Z., Zhang Z., Xu Y. (2022). Random color filters based on an all-dielectric metasurface for compact hyperspectral imaging. Opt. Lett..

[69] Zhao J., Yu X., Zhou K., Yang X., Yu Y. (2019). Wide-gamut and polarization-independent structural color at optical sub-diffraction-limit spatial resolution based on uncoupled lspps. Nanoscale Res. Lett..

[70] Jiang M., Siew S. Y., Chan J. Y. (2020). Patterned resist on flat silver achieving saturated plasmonic colors with sub-20-nm spectral linewidth. Mater. Today.

[71] Shi L., Niu J., Li L. (2022). Deep subwavelength wide-angle structural colors at the single pixel level. Adv. Opt. Mater..

[72] Zhu T., Wu T., Liu Y. (2019). All-dielectric colored truncated cone metasurfaces with silicon mie magnetic resonators. Appl. Opt..

[73] Joo W. J., Kyoung J., Esfandyarpour M. (2020). Metasurface-driven oled displays beyond 10,000 pixels per inch. Science.

[74] Yang J. H., Babicheva V. E., Yu M. W., Lu T. C., Lin T. R., Chen K. P. (2020). Structural colors enabled by lattice resonance on silicon nitride metasurfaces. ACS Nano.

[75] Yang W., Xiao S., Song Q. (2020). All-dielectric metasurface for high-performance structural color. Nat. Commun..

[76] Greybush N. J., Charipar K., Geldmeier J. A. (2019). Dynamic plasmonic pixels. ACS Nano.

[77] Song M., Feng L., Huo P. (2022). Versatile full-colour nanopainting enabled by a pixelated plasmonic metasurface. *Nat. Nanotechnol.*.

[78] Yang B., Liu W., Li Z. (2019). Ultrahighly saturated structural colors enhanced by multipolar-modulated metasurfaces. Nano Lett..

[79] Liu X., Huang Z., Zang J. (2020). All-dielectric silicon nanoring metasurface for full-color printing. Nano Lett..

[80] Yang B., Liu W., Choi D. Y. (2021). High-performance transmission structural colors generated by hybrid metal-dielectric metasurfaces. Adv. Opt. Mater..

[81] Peng J., Jeong H. H., Lin Q. (2019). Scalable electrochromic nanopixels using plasmonics. Sci. Adv..

[82] Rezaei S. D., Dong Z., Wang H. (2022). Tri-functional metasurface enhanced with a physically unclonable function. Mater. Today.

[83] Mueller J. B., Rubin N. A., Devlin R. C., Groever B., Capasso F. (2017). Metasurface polarization optics: independent phase control of arbitrary orthogonal states of polarization. Phys. Rev. Lett..

[84] Jiang Q., Jin G., Cao L. (2019). When metasurface meets hologram: principle and advances. Adv. Opt. Photonics.

[85] Huang L., Zhang S., Zentgraf T. (2018). Metasurface holography: from fundamentals to applications. Nanophotonics.

[86] Zhao R., Huang L., Wang Y. (2020). Recent advances in multi-dimensional metasurfaces holographic technologies. PhotoniX.

[87] Fan Q., Liu M., Zhang C. (2020). Independent amplitude control of arbitrary orthogonal states of polarization via dielectric metasurfaces. Phys. Rev. Lett..

[88] Song Q., Khadir S., Vézian S. (2020). Printing polarization and phase at the optical diffraction limit: near-and far-field optical encryption. Nanophotonics.

[89] Deng L., Deng J., Guan Z. (2020). Malus-metasurface-assisted polarization multiplexing. Light: Sci. Appl..

[90] Ren R., Li Z., Deng L. (2021). Non-orthogonal polarization multiplexed metasurfaces for tri-channel polychromatic image displays and information encryption. Nanophotonics.

[91] Zhao R., Xiao X., Geng G. (2021). Polarization and holography recording in real-and k-space based on dielectric metasurface. Adv. Funct. Mater..

[92] Mao N., Deng J., Zhang X. (2020). Nonlinear diatomic metasurface for real and fourier space image encoding. Nano Lett..

[93] Bao Y., Wen L., Chen Q., Qiu C.-W., Li B. (2021). Toward the capacity limit of 2d planar jones matrix with a single-layer metasurface. Sci. Adv..

[94] Bao Y., Nan F., Yan J., Yang X., Qiu C. W., Li B. (2022). Observation of full-parameter jones matrix in bilayer metasurface. Nat. Commun..

[95] Xiong B., Liu Y., Xu Y. (2023). Breaking the limitation of polarization multiplexing in optical metasurfaces with engineered noise. Science.

[96] Kim I., Kim W. S., Kim K. (2021). Holographic metasurface gas sensors for instantaneous visual alarms. Sci. Adv..

[97] Kim I., Jang J., Kim G. (2021). Pixelated bifunctional metasurface-driven dynamic vectorial holographic color prints for photonic security platform. Nat. Commun..

[98] Zheng P., Dai Q., Li Z. (2021). Metasurface-based key for computational imaging encryption. Sci. Adv..

[99] Guo X., Li P., Zhong J. (2022). Stokes meta-hologram toward optical cryptography. Nat. Commun..

[100] Cai T., Wang G., Tang S. (2017). High-efficiency and full-space manipulation of electromagnetic wave fronts with metasurfaces. Phys. Rev. Appl..

[101] Zhang L., Wu R. Y., Bai G. D. (2018). Transmission-reflection-integrated multifunctional coding metasurface for full-space controls of electromagnetic waves. Adv. Funct. Mater..

[102] Cai T., Tang S., Wang G. (2017). High-performance bifunctional metasurfaces in transmission and reflection geometries. Adv. Opt. Mater..

[103] Mao R., Wang G., Cai T., Liu K., Wang D., Wu B. (2020). Ultra-thin and high-efficiency full-space pancharatnam-berry metasurface. Opt. Express.

[104] Dolan J. A., Cai H., Delalande L. (2021). Broadband liquid crystal tunable metasurfaces in the visible: liquid crystal inhomogeneities across the metasurface parameter space. ACS Photonics.

[105] Li K., Wang J., Cai W. (2021). Electrically switchable, polarization-sensitive encryption based on aluminum nanoaperture arrays integrated with polymer-dispersed liquid crystals. Nano Lett..

[106] Zheng P., Li J., Li Z. (2022). Compressive imaging encryption with secret sharing metasurfaces. Adv. Opt. Mater..

[107] Georgi P., Wei Q., Sain B. (2021). Optical secret sharing with cascaded metasurface holography. Sci. Adv..

[108] Wei Q., Huang L., Zhao R. (2022). Rotational multiplexing method based on cascaded metasurface holography. Adv. Opt. Mater..

[109] Wan W., Yang W., Feng H. (2021). Multiplexing vectorial holographic images with arbitrary metaholograms. Adv. Opt. Mater..

[110] Deng J., Li Z., Li J. (2022). Metasurface-assisted optical encryption carrying camouflaged information. Adv. Opt. Mater..

[111] Wen D., Crozier K. B. (2021). Metasurfaces 2.0: laser-integrated and with vector field control. APL Photonics.

[112] Song Q., Khadir S., Vézian S. (2021). Bandwidth-unlimited polarization-maintaining metasurfaces. Sci. Adv..

[113] Rubin N. A., Zaidi A., Dorrah A. H., Shi Z., Capasso F. (2021). Jones matrix holography with metasurfaces. Sci. Adv..

[114] Dorrah A. H., Rubin N. A., Zaidi A., Tamagnone M., Capasso F. (2021). Metasurface optics for on-demand polarization transformations along the optical path. Nat. Photonics.

[115] Fan Q., Xu W., Hu X. (2022). Trilobite-inspired neural nanophotonic light-field camera with extreme depth-of-field. Nat. Commun..

[116] Arbabi E., Kamali S. M., Arbabi A., Faraon A. (2018). Full-Stokes imaging polarimetry using dielectric metasurfaces. ACS Photonics.

[117] Rubin N. A., D’Aversa G., Chevalier P., Shi Z., Chen W. T., Capasso F. (2019). Matrix fourier optics enables a compact full-Stokes polarization camera. Science.

[118] Sun T., Hu J., Zhu X., Xu F., Wang C. (2022). Broadband single-chip full Stokes polarization-spectral imaging based on all-dielectric spatial multiplexing metalens. Laser Photonics Rev..

[119] Intaravanne Y., Chen X. (2020). Recent advances in optical metasurfaces for polarization detection and engineered polarization profiles. Nanophotonics.

[120] Rubin N. A., Chevalier P., Juhl M., Tamagnone M., Chipman R., Capasso F. (2022). Imaging polarimetry through metasurface polarization gratings. Opt. Express.

[121] Devlin R. C., Ambrosio A., Rubin N. A., Mueller J. B., Capasso F. (2017). Arbitrary spin-to–orbital angular momentum conversion of light. Science.

[122] Sroor H., Huang Y. W., Sephton B. (2020). High-purity orbital angular momentum states from a visible metasurface laser. Nat. Photonics.

[123] Bao Y., Ni J., Qiu C. W. (2020). A minimalist single-layer metasurface for arbitrary and full control of vector vortex beams. Adv. Mater..

[124] Dorrah A. H., Rubin N. A., Tamagnone M., Zaidi A., Capasso F. (2021). Structuring total angular momentum of light along the propagation direction with polarization-controlled meta-optics. Nat. Commun..

[125] Li T., Xu X., Fu B. (2021). Integrating the optical tweezers and spanner onto an individual single-layer metasurface. Photonics Res..

[126] Fang X., Ren H., Gu M. (2020). Orbital angular momentum holography for high-security encryption. Nat. Photonics.

[127] Ren H., Fang X., Jang J., Bürger J., Rho J., Maier S. A. (2020). Complex-amplitude metasurface-based orbital angular momentum holography in momentum space. Nat. Nanotechnol..

[128] Liu S. J., Chen P., Ge S. J., Zhu L., Zhang Y. H., Lu Y. Q. (2022). 3d engineering of orbital angular momentum beams via liquid-crystal geometric phase. Laser Photonics Rev..

[129] Ren H., Briere G., Fang X. (2019). Metasurface orbital angular momentum holography. Nat. Commun..

[130] Kamali S. M., Arbabi E., Arbabi A., Horie Y., Faraji-Dana M., Faraon A. (2017). Angle-multiplexed metasurfaces: encoding independent wavefronts in a single metasurface under different illumination angles. Phys. Rev. X.

[131] Zhang X., Li Q., Liu F. (2020). Controlling angular dispersions in optical metasurfaces. Light: Sci. Appl..

[132] Zhang X., Jin J., Pu M. (2017). Ultrahigh-capacity dynamic holographic displays via anisotropic nanoholes. Nanoscale.

[133] Wan S., Wan C., Dai C. (2021). Angular-multiplexing metasurface: building up independent-encoded amplitude/phase dictionary for angular illumination. Adv. Opt. Mater..

[134] Jang J., Lee G. Y., Sung J., Lee B. (2021). Independent multichannel wavefront modulation for angle multiplexed meta-holograms. Adv. Opt. Mater..

[135] Frese D., Wei Q., Wang Y., Huang L., Zentgraf T. (2019). Nonreciprocal asymmetric polarization encryption by layered plasmonic metasurfaces. Nano Lett..

[136] Chen Y., Yang X., Gao J. (2019). 3d janus plasmonic helical nanoapertures for polarization-encrypted data storage. Light: Sci. Appl..

[137] Shalaginov M. Y., An S., Yang F. (2020). Single-element diffraction-limited fisheye metalens. Nano Lett..

[138] Hao C., Gao S., Ruan Q. (2020). Single-layer aberration-compensated flat lens for robust wide-angle imaging. Laser Photonics Rev..

[139] Yoon G., Lee D., Nam K. T., Rho J. (2018). crypto-display” in dual-mode metasurfaces by simultaneous control of phase and spectral responses. ACS Nano.

[140] Chen K., Ding G., Hu G. (2020). Directional janus metasurface. Adv. Mater..

[141] Kim T., Yu E. S., Bae Y. G. (2020). Asymmetric optical camouflage: tuneable reflective colour accompanied by the optical janus effect. Light: Sci. Appl..

[142] Chu H., Xiong X., Gao Y. J. (2021). Diffuse reflection and reciprocity-protected transmission via a random-flip metasurface. Sci. Adv..

[143] Arbabi A., Arbabi E., Kamali S. M., Horie Y., Han S., Faraon A. (2016). Miniature optical planar camera based on a wide-angle metasurface doublet corrected for monochromatic aberrations. Nat. Commun..

[144] Groever B., Chen W. T., Capasso F. (2017). Meta-lens doublet in the visible region. Nano Lett..

[145] Zhang F., Pu M., Li X. (2021). Extreme-angle silicon infrared optics enabled by streamlined surfaces. Adv. Mater..

[146] Liu W., Li Z., Cheng H. (2018). Metasurface enabled wide-angle fourier lens. Adv. Mater..

[147] Martins A., Li K., Li J. (2020). On metalenses with arbitrarily wide field of view. ACS Photonics.

[148] Lassalle E., Mass T. W., Eschimese D. (2021). Imaging properties of large field-of-view quadratic metalenses and their applications to fingerprint detection. ACS Photonics.

[149] Chen J., Ye X., Gao S. (2022). Planar wide-angle-imaging camera enabled by metalens array. Optica.

[150] Li S., Hsu C. W. (2022). Thickness bound for nonlocal wide-field-of-view metalenses. Light: Sci. Appl..

[151] Tan Q., Zheng B., Cai T. (2022). Broadband spin-locked metasurface retroreflector. Adv. Sci..

[152] Kim G., Kim Y., Yun J. (2022). Metasurface-driven full-space structured light for three-dimensional imaging. Nat. Commun..

[153] Leitis A., Tittl A., Liu M. (2019). Angle-multiplexed all-dielectric metasurfaces for broadband molecular fingerprint retrieval. Sci. Adv..

[154] Shi Z., Zhu A. Y., Li Z. (2020). Continuous angle-tunable birefringence with freeform metasurfaces for arbitrary polarization conversion. Sci. Adv..

[155] Arbabi A., Arbabi E., Horie Y., Kamali S. M., Faraon A. (2017). Planar metasurface retroreflector. Nat. Photonics.

[156] Martins R. J., Marinov E., Youssef M. A. B. (2022). Metasurface-enhanced light detection and ranging technology. Nat. Commun..

[157] Feng H., Li Q., Wan W. (2019). Spin-switched three-dimensional full-color scenes based on a dielectric meta-hologram. ACS Photonics.

[158] Li N., Fu Y. H., Dong Y. (2019). Large-area pixelated metasurface beam deflector on a 12-inch glass wafer for random point generation. Nanophotonics.

[159] Liu Z., Zhang C., Zhu W. (2021). Compact stereo waveguide display based on a unidirectional polarization-multiplexed metagrating in-coupler. ACS Photonics.

[160] Shi Y., Wan C., Dai C. (2022). On-chip meta-optics for semi-transparent screen display in sync with ar projection. Optica.

[161] Song J. H., van de Groep J., Kim S. J., Brongersma M. L. (2021). Non-local metasurfaces for spectrally decoupled wavefront manipulation and eye tracking. Nat. Nanotechnol..

[162] Kim I., Martins R. J., Jang J. (2021). Nanophotonics for light detection and ranging technology. Nat. Nanotechnol..

[163] Ni Y., Chen S., Wang Y., Tan Q., Xiao S., Yang Y. (2020). Metasurface for structured light projection over 120 field of view. Nano Lett..

[164] Li Z., Pestourie R., Park J. S., Huang Y. W., Johnson S. G., Capasso F. (2022). Inverse design enables large-scale high-performance meta-optics reshaping virtual reality. Nat. Commun..

[165] Tian Y., Tang H., Kang T., Guo X., Wang J., Zang J. (2022). Inverse-designed aid lenses for precise correction of color vision deficiency. Nano Lett..

[166] Qu G., Yang W., Song Q. (2020). Reprogrammable meta-hologram for optical encryption. Nat. Commun..

[167] de Galarreta C. R., Sinev I., Alexeev A. M. (2020). Reconfigurable multilevel control of hybrid all-dielectric phase-change metasurfaces. Optica.

[168] Shalaginov M. Y., An S., Zhang Y. (2021). Reconfigurable all-dielectric metalens with diffraction-limited performance. Nat. Commun..

